# Integrated gradient tissue-engineered osteochondral scaffolds: Challenges, current efforts and future perspectives

**DOI:** 10.1016/j.bioactmat.2022.06.011

**Published:** 2022-07-01

**Authors:** Xiaolian Niu, Ning Li, Zhipo Du, Xiaoming Li

**Affiliations:** a Key Laboratory for Biomechanics and Mechanobiology of Ministry of Education, Beijing Advanced Innovation Center for Biomedical Engineering, School of Biological Science and Medical Engineering, Beihang University, Beijing, 100083, China; b Department of Orthopedics, The Fourth Central Hospital of Baoding City, Baoding, 072350, China

**Keywords:** Osteochondral tissue engineering, Integrated gradient tissue-engineered osteochondral scaffold (IGTEOS), Tissue-engineered strategies, Fabrication techniques, Evaluation

## Abstract

The osteochondral defect repair has been most extensively studied due to the rising demand for new therapies to diseases such as osteoarthritis. Tissue engineering has been proposed as a promising strategy to meet the demand of simultaneous regeneration of both cartilage and subchondral bone by constructing integrated gradient tissue-engineered osteochondral scaffold (IGTEOS). This review brought forward the main challenges of establishing a satisfactory IGTEOS from the perspectives of the complexity of physiology and microenvironment of osteochondral tissue, and the limitations of obtaining the desired and required scaffold. Then, we comprehensively discussed and summarized the current tissue-engineered efforts to resolve the above challenges, including architecture strategies, fabrication techniques and *in vitro*/*in vivo* evaluation methods of the IGTEOS. Especially, we highlighted the advantages and limitations of various fabrication techniques of IGTEOS, and common cases of IGTEOS application. Finally, based on the above challenges and current research progress, we analyzed in details the future perspectives of tissue-engineered osteochondral construct, so as to achieve the perfect reconstruction of the cartilaginous and osseous layers of osteochondral tissue simultaneously. This comprehensive and instructive review could provide deep insights into our current understanding of IGTEOS.

## Highlights

• Providing main challenges to establish integrated gradient osteochondral scaffold.
• Discussing the current tissue-engineered efforts to resolve the above challenges.
• Highlighting construct techniques, and evaluation index and methods of IGTEOS.
• Discussing the future perspectives to achieve perfect osteochondral reconstruction.

## Graphical abstract

**Figure:**
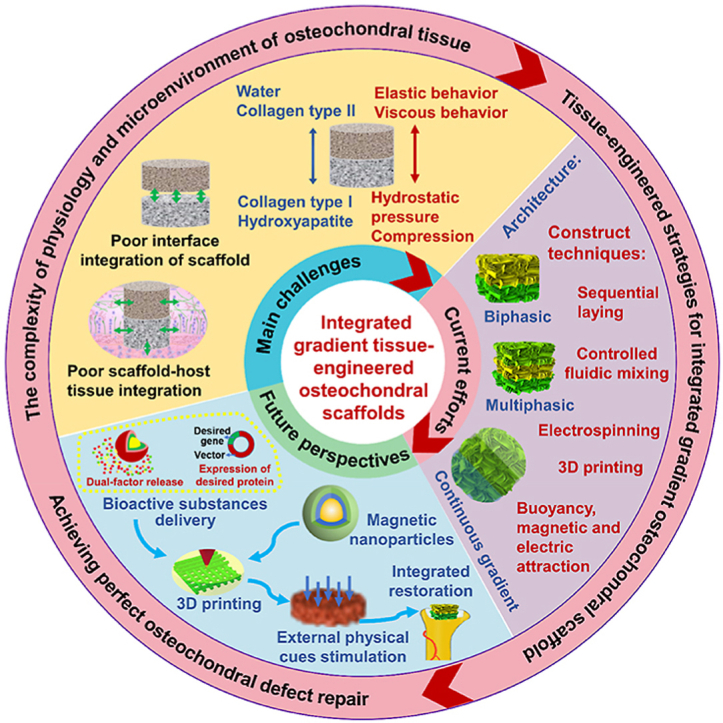

## 1 Introduction

**Fig. 1:**
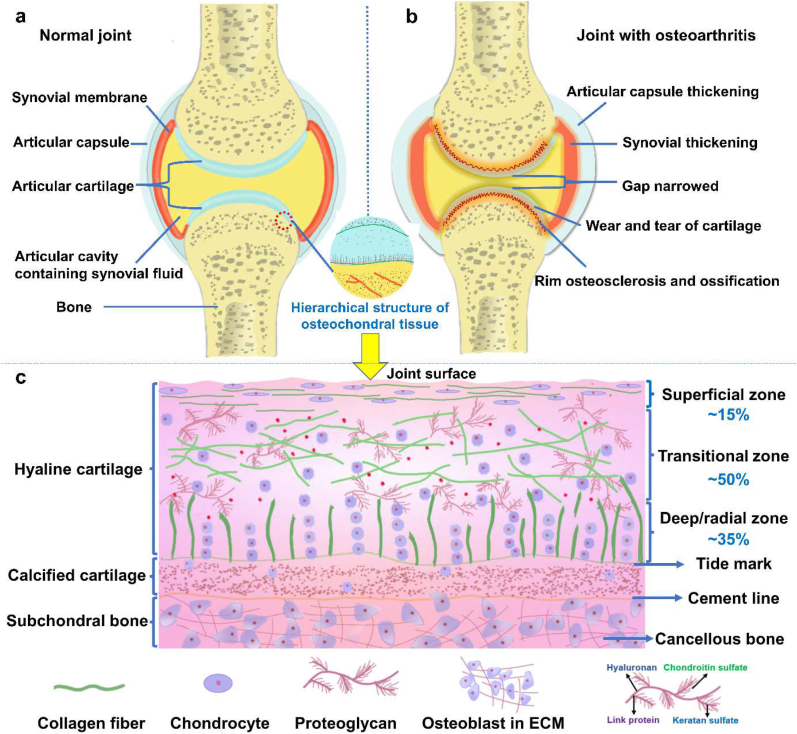
The schematic diagram of (a) normal joint, (b) diseased joint, and (c) osteochondral unit including cartilage, calcified cartilage and subchondral bone.

Osteochondral defects refer to the damage of cartilage as well as subchondral bone [1], which usually derive from traumatic injuries, inflammation, osteochondritis dissecans or chondromalacia. As shown in Fig. 1a, cartilage adheres to subchondral bone via a specific osteochondral interface tissue where forces are transferred from soft cartilage to hard bone, thereby preventing fatigue damage over a lifetime of load cycles [2]. Osteochondral defects are often associated with the mechanical instability of the joint, leading to osteoarthritis (Fig. 1b), which have affected the health of millions of people worldwide and caused a severe socio-economic burden to society [3,4].

The native osteochondral interface tissue connects two other tissues with different structure, chemical compositions and mechanical properties, and possesses complex physiological properties and gradient variation in structure, composition and function (Fig. 1c) [5]. The extracellular matrix (ECM) throughout cartilage tissue is itself secreted and modulated by the encapsulated chondrocytes, presenting a complex gradient of biochemical signal [6]. Cells interact with these stimuli in a spatiotemporal manner, via integrins and other cell-surface receptors, activating biological responses such as cell migration, proliferation, differentiation and apoptosis. IGTEOS that mimics the hierarchical nature of native osteochondral ECM has presented a sustainable and effective treatment for osteochondral defects [7,8].

This review aims to provide a comprehensive overview of IGTEOS, including main challenges, current efforts and future perspectives. Therefore, this review began with a detailed introduction of difficulties of osteochondral regeneration, traditional therapies and main challenges of establishing tissue-engineered osteochondral scaffolds, followed by discussion of tissue-engineered strategies of the IGTEOS, such as requirements of osteochondral scaffolds, architecture strategies, and selection of seed cells and growth factor, to give the readers a clear picture of osteochondral tissue engineering. Moreover, we extensively reviewed the fabrication techniques of IGTEOS and emphasized their advantages and limitations respectively. Furthermore, we discussed the evaluation methods of IGTEOS by focusing on *in vivo* and *in vitro* evaluation. Finally, before a brief summary, we also offered perspectives of tissue-engineered osteochondral construct on the challenges, opportunities and new directions for future development.

## 2 Challenge of osteochondral regeneration

**Fig. 2:**
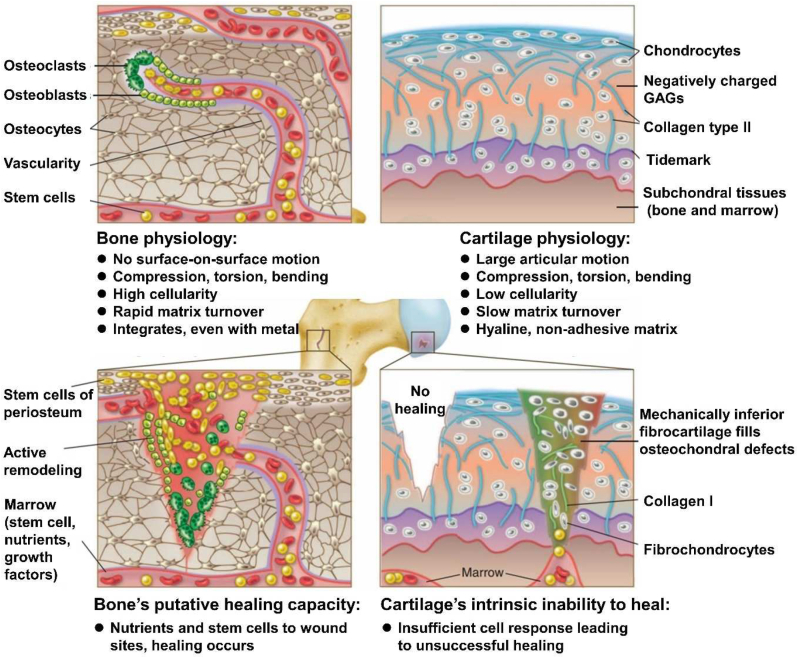
The schematic diagram illustrated the difference in the physiologic environment and healing capacities of cartilage and bone tissue. Reproduced with permission [5]: copyright 2012, AAAS.

Osteochondral tissue has a thickness of approximately 3 mm in adults, which is composed of cartilage, a calcified cartilage layer, and the subchondral bone in a proportion of 90%, 5%, and 5%, respectively [9]. The complexity of cartilage-bone interface and the dissimilar healing capacities between cartilage and subchondral bone layers particularly impede successful regenerative treatment of osteochondral lesion. Differences in the physiologic environment, biomechanical properties, metabolic rate and cellular composition of bone and cartilage have profound effects on the osteochondral regeneration (Fig. 2). Specifically, the main challenges of osteochondral regeneration be summarized as the following aspects: 1) The cartilage and bone with dissimilar healing capacities. The periosteum and bone marrow contain stem cells that could differentiate into bone-producing cells, and a large number of osteoclasts and osteoblasts are involved in perpetual bone breakdown and remodeling [5]. Moreover, bone's extensive vascularity provides abundant nutrients and blood-borne proteins which could stimulate tissue self-repaired up to a critical size. For the large bone defects requiring vascularization, bone repair employing *in situ* mesenchymal stem cells (MSCs) could be augmented by osteoconductive or osteoinductive scaffolds with or without growth factors [10]. However, articular cartilage has a poor self-reparative capacity after injury or degenerative diseases due to its avascular characteristics, low cellularity and the poor chondrocytes proliferation ability, which makes cartilage repair facing great challenges in clinics [11]. 2) In terms of integration, the adhesive nature of hyaline cartilage precludes integration, while bone integration is rapid [5]. 3) Despite above fact, the tissues in the osteochondral unit coexist as a single functional unit during both physiological and pathological conditions where exists a close interaction between cartilage and bone [12]. Articular cartilage is essential for articulation of load-bearing joints and serves to distribute load, absorb shock and facilitate motion [13]. Therefore, compared to bone's healing ability, cartilage requires a more robust exogenous approach to achieve satisfactory regeneration. 4) Moreover, natural osteochondral systems frequently exhibit gradients along different structural axes and show dynamic changes in morphogen gradient profiles during different stages of development, which are also major challenges for biomaterial-based strategies.

### 2.1 Current clinical treatment strategies for osteochondral defects

Currently, surgical procedure is a common approach to treat osteochondral injury, which could be divided into palliative, reparative and restorative treatments according to the level of repair they provide to the osteochondral defect site [14]. Palliative approaches, including arthroscopic debridement, abrasion arthroplasty and chondroplasty, provide symptomatic relief rather than replacing defective osteochondral tissue. Reparative strategies include microfracture and drilling, as well as autologous or allogeneic osteochondral transplantation (mosaicplasty), aiming to repair or regenerate damaged osteochondral tissue [15]. Nevertheless, due to the specific nature of osteochondral tissue, the ideal repair method is a restorative treatment method that helps to reconstruct the natural tissue. Restorative tactics, such as autologous chondrocyte implantation (ACI) and matrix-induced autologous chondrocyte implantation (MACI), utilize osteochondral or chondrocyte transplantation to repair or regenerate damaged osteochondral tissue [16]. Currently ACI is the only truly restorative clinical treatment method for cartilage damage. ACI technique involves biopsy material harvesting, followed by the implantation of cultured autologous chondrocytes into the debrided defect area and covered with a periosteal flap. This technique could avoid potential immune complications from transplanting allogeneic cells or foreign materials, and the small biopsy minimizes complications for the chondrocyte donor [17]. Based on the ACI methods, autologously isolated and enriched chondrocytes are seeded on a synthetic matrix and then implanted onto the defect site without the use of a periosteal flap---this is termed MACI [18], which is the most common scaffold-cell-based cartilage repair technique currently in clinical practice. The 3D supporting matrix could be optimized from both the biological and surgical point of view, as it helps to evenly distribute chondrocytes at the defect site and avoids the need for highly invasive procedures. Nevertheless, the limitations of ACI and MACI include the requirement for two surgical procedures, typically rather invasive, and relatively long recovery time to ensure neo-tissue maturation, which hamper their wide application in the osteochondral therapy [17].

To improve the efficacy of such tissue engineering procedures, surgical techniques similar to those established for MACI are used, during which autologous chondrocytes are introduced into a 3D matrix and cultured *in vitro* for longer periods, and then articular chondrocytes produce their own ECM components within the 3D environment, resulting in an implant with biochemical integrity similar to healthy articular cartilage [17,19]. However, time alone is not enough to promote sufficient maturation of the engineered tissue. Exogenous mechanical stimulations including hydrostatic pressure and dynamic compression have been applied to cell-laden matrices *in vitro*, to improve matrix maturation and the function of neo-tissues [[20], [21], [22], [23], [24]]. To further promote sufficient maturation of the engineered tissue, researchers have developed new strategies to combine both growth factors and MSCs chemokines by tailoring their release in a controlled manner, for example on the surface of matrices or within nanoparticles [[25], [26], [27], [28]]. The osteochondral tissue engineering combines seed cells, growth factors and 3D scaffolds to form a seamless transition from hard to soft tissues [29], during which each layer of the scaffold should be engineered according to tissue-specific biophysical conditions and microenvironments to support a unique cell type, achieving complete osteochondral regeneration. Even in cases where lesion does not penetrate to the subchondral bone, an osteochondral construct may be a more ideal implant, as a bone-to-bone interface integration is superior to a cartilage-to-cartilage interface [30].

### 2.2 Limitations of making an engineered osteochondral construct for clinical use

**Fig. 3:**
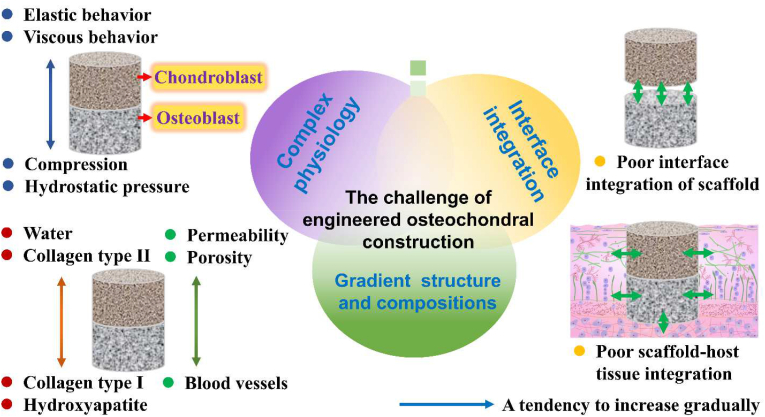
Challenges of making an integrated gradients tissue-engineered osteochondral construct for clinical use, including complex physiology, interface integration, and gradient structure and composition.

There are grand challenges still exist in the osteochondral defect repair due to the complexity of the osteochondral tissue and the high clinical demand for interface tissue [31,32]. 1) The osteochondral ECM is characterized by gradual changes in composition, mechanics and structure from bone to cartilage, where collagen type II (Col-II) and water content increase whereas mineral storage decreases [33]. In addition, the compressive modulus, porosity and pore size of osteochondral ECM decrease from bone to cartilage [34]. These smooth variations between vascular/mineralized bone and non-vascular/non-mineralized cartilage are critical for maintaining cartilage-to-bone stability. 2) Osteochondral scaffolds should be designed to restore the defect of cartilage, intermediate calcified cartilage and bone tissues concurrently. Therefore, cartilage component should be a hydrated viscoelastic matrix with relatively low compressive modulus, while osseous component should be a vascularized stiff framework with high modulus [35]. 3) The low interfacial bonding between the cartilaginous layer and bony layer of osteochondral scaffold, and the poor integration of engineered osteochondral with host tissues are still two main problems for tissue-engineered osteochondral scaffold [3,13]. 4) The disease status of individuals also establishes a major challenge in fabricating engineering scaffolds that will meet the demand of specific repair sites in specific patients. The main challenges of making an engineered osteochondral construct for clinical use are schematically outlined in Fig. 3. For osteochondral defects, the concurrent treatments of injured cartilage and subchondral bone need IGTEOS to support simultaneous reconstruction of both tissue phases. In particular, hierarchical scaffolds have been amalgamated together via the integration of a mutual material common to both layers [29], which could provide a complete transition between the bone and cartilage scaffold layers without requiring a joining procedure during implantation. In recent studies, IGTEOS has been fabricated by combining different additive manufacturing techniques and other methods [36].

## 3 Osteochondral tissue engineering

**Fig. 4:**
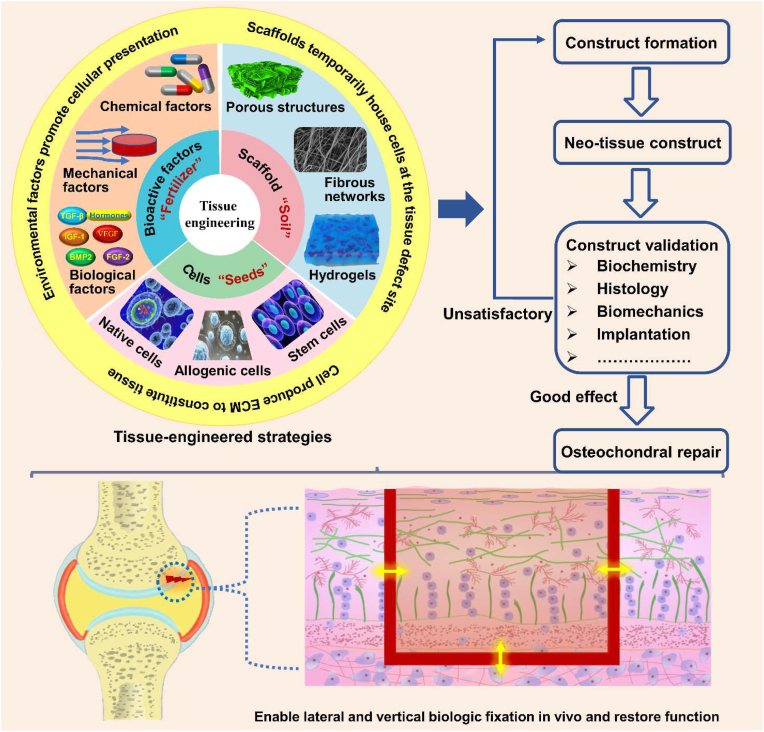
The building process of a tissue-engineered osteochondral construct: tissue-engineered osteochondral strategies usually resort to the combination of innovative biomaterials, cells and signal molecule, aiming to recapitulate the biological, physical and functional features of the native osteochondral unit; after repeated evaluation and validation, such biomimicking constructs could then be implanted into a damaged osteochondral region, where they will assist tissue repair, promote regenerative responses and facilitate the functional recovery of the joint.

Tissue engineering combines the knowledge of cells, engineering materials, and biochemical factors for the development of biological substitutes that restore, maintain, or regenerate the damaged tissues to improve tissue function [37,38]. The paradigm of *in vitro* osteochondral tissue engineering is showed in Fig. 4. The scaffold is a temporary structural support that mimics the osteochondral ECM and also serves as a temporary matrix for cell attachment, proliferation and differentiation to reconstruct damaged osteochondral tissues [39]. The cell synthesizes new tissue, while bioactive factors and drugs facilitate and promote cells to regenerate new tissue. The successful design of cell-instructive microenvironment requires consideration of general biological and physical criteria, as well as specific tissue characteristics. In the following section, we briefly highlighted the architecture strategy of osteochondral scaffolds, overviewed their composition, and illustrated the selection of cell sources and signaling molecules. All of those are considered to be significant elements in engineering functional tissues and mimicking tissue-equivalents.

### 3.1 Design of scaffolds

**Fig. 5:**
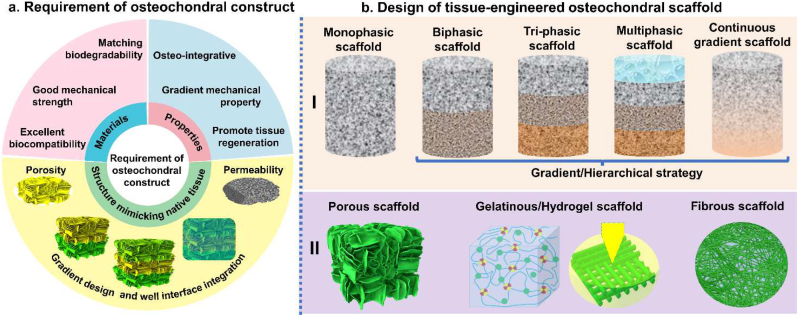
Tissue-engineered strategies of osteochondral scaffold. (a) Requirement of integrated gradient tissue-engineered osteochondral construct: the materials of osteochondral scaffold should have matching biodegradability, good mechanical strength and excellent biocompatibility; the structure of osteochondral scaffold should mimic native tissue, including suitable pore sizes and porosity, gradient design and well interface integration; some properties of osteochondral scaffold are essential, such as good osseointegration, gradient mechanical property and improved tissue regeneration. (b) Schematic diagram of design of tissue-engineered osteochondral scaffold *in vitro*: I) Scaffold strategies could be classified according to the number of layers and gradient properties of the designs; II) Micromorphology of osteochondral scaffold.

It is well known that the osteochondral scaffold should provide a 3D gradient structure, suitable porosity, matching biodegradability, good biocompatibility, initial mechanical strength and osteo-integration [40,41]. Considering the concurrent treatment of injured cartilage and subchondral bone, IGTEOS that mimics the hierarchical nature of native osteochondral ECM should support simultaneous reconstruction of both tissue phases, presenting a sustainable and effective treatment for osteochondral defects [42,43]. The requirements of osteochondral scaffold are shown in Fig. 5a. Lien et al. [44] reported that scaffolds with pore diameters of 200–500 μm supported efficient proliferation and distribution of chondrocytes. *In vitro* and *in vivo* experimental tests demonstrated that scaffolds with pore diameters of 100–500 μm were optimal for bone regeneration [45,46]. Relatively larger pores facilitated direct osteogenesis, since they allowed vascularization and high oxygenation, while smaller pores resulted in osteochondral ossification [47]. However, an increase in scaffold porosity could greatly diminish the mechanical properties, preventing the structure from performing essential load-bearing responsibilities [48]. Considering the mechanical properties requirement, the pore size of osteochondral scaffold depends on many factors, including the nature of the biomaterial and the processing conditions used to fabricate the 3D scaffold. The ideal biocompatible IGTEOS should promote the establishment of a calcified cartilage matrix with physiologically relevant mechanical properties. The initial mechanical strength and osseointegration could guarantee its function as temporary matrix for tissue growth. Moreover, the zonal organization and zone-specific cellular phenotype of osteochondral tissue have been developed by regulating the secretion and spatial distribution of the bioactive factors [49,50]. Therefore, the incorporation of gradient cellular signals into scaffolds in a spatially controlled way could facilitate regional regulation of cell for engineering biomimetic tissues. Furthermore, the scaffolds biodegradability should be match with the formation rate of the neo-tissue, to ensure that cells have time to synthesize their own ECM and produce functional neo-tissues [51]. The following section focused on the composition and architecture strategy of IGTEOS, including monophasic scaffold, biphasic scaffold, triphasic and multilayered scaffolds, and continuous gradient scaffolds.

#### 3.1.1 Composition

Considering the composition of IGTEOS, the scaffold materials are divided into following categories, such as natural biomaterial, synthetic material, biological ceramics, and ECM-based and composite material [52]. As shown in Table 1, we have summarized the advantage and disadvantage of each category of materials, and listed some specific materials for IGTEOS. For natural biomaterials, the cellular compatibility and bioactivity are generally superior to synthetic polymers, but mechanical properties are relatively weak, and the degradation rate is difficult to control. While synthetic.

**Table 1:** Common materials of osteochondral scaffold.

Advantage/Disadvantage	Materials name	Ref
Natural biomaterial		
**Advantage:** Containing bioactive factors that may promote desirable cellular functions such as cell adhesion, proliferation and differentiation **Disadvantage:** Batch-to-batch variability, the possibility of pathogen transfer, poor mechanical properties, and limited control over physiochemical properties	Collagen (Col)	[[53], [54], [55]]
	Gelatin (Gel)	[56,57]
	Peptides	[58,59]
	Hyaluronic acid (HAc)	[29,54,60]
	Alginate (SA)	[61,62]
	Agarose (AG)	[63]
	Cellulose, Bacterial cellulose (BC)	[61,64]
	Chitosan (CS)	[65,66]
	Fibrinogen (Fg)	[67]
	Silk fibroin (SF)	[57,58,63,66,[68], [69], [70]]
**Synthetic material**		
**Advantage:** The physiochemical and mechanical properties could be modulated during the synthesis process to suit various application **Disadvantage:** Lacking integrin-binding ligands limited its inherent interaction with cells	Polyethylene glycol (PEG)	[65]
	Polycaprolactone (PCL)	[7,[71], [72], [73]]
	Polylactic acid (PLA)	[[74], [75], [76], [77]]
	Poly (lactic-co-glycolic acid) (PLGA)	[78,79]
	Gelatin methacrylate (GelMA)	[[80], [81], [82]]
	Poly (vinyl alcohol) (PVA)	[62]
	Poly (N-isopropyl acrylamide) (PNIPAAm)	[60]
	Poly (ethylene oxide) (PEO)	[83]
	Polyacrylamide (PAAM)	[84]
	Poly (ethylene glycol) diacrylate (PEGDA)	[85]
**Biological ceramics**		
**Advantage:** Integrated well with the bone tissue and possess superior osteoconductive properties **Disadvantage:** Brittle and slow to degrade	Bioactive glass (BG)	[86]
	Hydroxyapatite (HA)	[54,55,62,65,68,71,73,81,87]
	Biphasic calcium phosphate (BCP)	[88]
	Tricalcium phosphate (TCP)	[78,89]
	Nano-silicate	[67,69,90,91]
**Extracellular matrix**		
**Advantage:** Retained spatial structure of ECM and growth factors in native tissue, and no immunogenicity **Disadvantage:** Elevated density of the matrix may hinder tissue remodel and graft integration	Cartilage extracellular matrix	[82,[92], [93], [94]]
	Demineralized bone powder (DBP)	[95,96]
	Decellularized extracellular matrix (dECM)	[97,98]

Materials have excellent flexibility to adapt their shape to required forms *via* various molding and casting techniques, but the poor surface activity and cell affinity, slow degradation rates and harmful degradation products restrict its application [99]. Beneficial molecules (such as GAGs, collagen and GAG-like polysaccharides) for cells are rich in natural biomaterials and ECM, and thus, these biomaterials could be introduced into synthetic materials so as to improve the biological affinity of the scaffold to the host tissue [52]. Inspired by the gradients in ECM composition and collagen fiber architecture in native osteochondral tissue, Qiao et al. [8] designed a stratified scaffold in which MSC-laden GelMA hydrogel with zone-specific growth factor delivery was combined with melt electro-written triblock polymer of poly(e-caprolactone) and poly (ethylene glycol) (PCEC) networks with depth-dependent fiber organization. Introducing PCEC fibers into the GelMA hydrogel contributed to a significant increase in mechanical strength.

Regarding material composition, and complex properties and functions of osteochondral scaffold, the upper cartilage layer favors hydrogels based on natural or synthetic polymers (owning to their hydrated nature and viscoelasticity are similar to the native ECM), the lower subchondral layer prefers reinforced materials such as bio-ceramics and harder polymers, and the intermedia layer (bone-cartilage interface) adopts the combination of chondral layer and bone layer materials with a specific proportion.

#### 3.1.2 Architecture strategy

To successfully construct an ideal scaffold for the regeneration of osteochondral tissue, the architecture is another crucial factor in addition to the material composition. Considering the structure forms of scaffold, the porous structures, fibrous networks and hydrogels are important candidates for repairing osteochondral defects, because they could accurately mimic the complexity of osteochondral units to facilitate the formation of new osteochondral tissue [100]. In order to generate a smooth transition between hard and stiff bone tissue and the softer and viscoelastic articular cartilage, it is necessary to mimic the anatomical and physicochemical properties of native osteochondral tissue as closely as possible to design gradient scaffolds. In the past years, IGTEOS has been developed from the simplest monophasic scaffolds to biphasic, triphasic and multiphasic ones, as schematically outlined in Fig. 5b. These scaffolds are characterized by different mechanical properties and spatial structures of different parts, and even different loading abilities of growth factors or cell. The construct techniques of IGTEO were described in detail in the following section.

##### 3.1.2.1 Monophasic scaffolds

Monophasic scaffolds, one of the first derived osteochondral repair techniques, are any singular material preformed according to the defect area. Some common materials used for monophasic scaffolds are hydroxyapatite (HA) or polymers which could be fabricated differently to achieve the ideal degradation rate, strength and porosity to properly mimic the properties of the native osteochondral tissue [101,102]. More specifically, a monophasic scaffold should contain the same materials in the same proportions throughout the scaffold, or a mixture of several materials, including a polymer combination or the addition of a gel phase throughout the pores of the scaffold [40]. Monophasic scaffolds support chondrocyte and bone cell attachment and proliferation. However, monophasic scaffolds lack the inherent physical structure and properties required to repair osteochondral tissue, and cannot simulate the biological environment well, so they are inadequate to replace defective osteochondral tissue.

##### 3.1.2.2 Biphasic scaffolds

The clinical success of mosaicplasty brings the idea of engineering biphasic osteochondral composites for osteochondral repair. Stratified scaffolds with distinct bone and cartilage phases in a single structure have been proposed as one of the most optimal osteochondral scaffolds, which characterized by gradient chemical composition, structure and mechanical properties. Compared with the monophasic scaffold, the hierarchical scaffold has the following advantages. 1) Hierarchical scaffolds could be optimized by adding appropriate growth factors to mimic cartilage and bone tissue separately. 2) Hierarchical scaffolds could be precultured for osteogenesis and chondrogenesis in a double-chamber bioreactor before the implantation *in vivo* [103]. 3) Hierarchical scaffolds could provide appropriate chemical, mechanical and biological stimulation that the tissue necessary for cell proliferation and/or differentiation. 4) Hierarchical scaffolds could give a suitable microenvironment to direct the communications between cell/cell and cell/matrix [104]. Numerous biphasic scaffolds have progressed into the preclinical animal studies and shown some degree of success, and a few are even commercially available for clinical utilization now [105]. However, as a primary determinant in maintaining the microenvironment of the two distinct tissues, the natural chondral-osseous interface (calcified cartilage) is ignored in biphasic scaffolds. In addition, biphasic scaffolds did not display all the gradients that characterize the osteochondral tissue.

##### 3.1.2.3 Triphasic and multilayered scaffolds

Given the fact that the osteochondral unit consists of hierarchical distinct zones with varying structures and compositions, triphasic and multilayered scaffolds involving the calcified cartilage simulation have been developed. The calcified cartilage is a narrow tissue layer that marks the transition from soft cartilage to stiff subchondral bone and contributes to the conversion of shear stresses into compressive and tensile stresses during joint loading and kinematics [106,107]. The introduction of transition layer not only acts as a physical barrier to inhibit vascular invasion into the cartilage to prevent the ossification of full-thickness cartilage, but also plays a role in supporting the load from the articular cartilage, which is beneficial for the integration of the implants with host tissues at the interface [108]. Compared with the biphasic scaffold, the scaffold with the compact intermediate layer fabricated from Poly (lactic-co-glycolic acid) (PLGA) and β-tricalcium phosphate (β-TCP) exhibited significantly higher anti-tensile and anti-shear properties as well as better *in vivo* regeneration results [109]. It is well known that osteochondral tissue has a distinctive hierarchical structure and biological properties which translate into unique biomechanical abilities [5]. Hence, triphasic scaffolds (only mimicking articular cartilage, calcified cartilage and subchondral bone) have difficulties meeting the full complexity of the chondro-osseous junction tissue, and so multilayered scaffolds with gradient physical and chemical properties are essential to produce smooth transitions between osteochondral tissues with significant differences. The triphasic and multilayered matrices with discrete gradient were fabricated by integrating individual phases into a single construct by suturing, gluing, and press-fitting [[110], [111], [112]]. It is important here to mention that there is no distinct interface between each layer of triphasic and multilayered scaffolds [110].

##### 3.1.2.4 Continuous gradient scaffolds

The native gradient and anisotropic structure in ECM deposition and cell type provide excellent permeability in deep zone (vessel ingrowth) and desired mechanical support [113]. Continuous transitions possess greater relevance to most natural systems, enabling improved load transmission and avoiding interfaces that could present mechanical instability. Sun et al. [114] concluded that biomimetic constructs mimicking the gradient anisotropic structure and the signaling approaches in different layers could induce zonal-dependent chondrogenic differentiation and ECM deposition. However, triphasic and multilayered scaffolds showed abrupt and substantial changes in terms of the structural and mechanical properties of the different phases, which was often associated with layer delamination and tissue separation upon loading [110,115]. The continuous gradient scaffolds did not exhibit individual layers and were fabricated as a single matrix with gradient properties [[110], [111], [112]]. The continuous gradient scaffolds have been developed by buoyancy, magnetic attraction and electric attraction techniques, in which a gradual transition between separate regions could better emulate the native features of the joint [[116], [117], [118]]. Those continuous gradient scaffolds are superior to monophasic and biphasic ones in regenerating osteochondral defects [62,119,120]. In addition, continuous gradient scaffolds are prepared as a single gradient matrix with gradient properties avoiding layer delamination and tissue separation upon loading, which could promote the chondrogenic and osteogenic differentiation of bone-marrow-derived mesenchymal stem cells (BMSCs) and ECM deposition.

Although the goal of tissue engineering is to achieve biomimicry, tissue-engineered approaches should also aim to create neo-tissue that withstands joint inflammation, readily integrates into surrounding native tissues and ensures positive outcomes regardless of biological variability and disease status of individuals. Further recapitulation of a native osteochondral tissue and creation of more complexity in osteochondral scaffolds will lead them to real-world clinical applications more powerfully. Clinical results from the current osteochondral scaffolds indicated that a multi-layered or hierarchical tissue-engineered approaches offer the most promising results with patients and their conditions [36].

### 3.2 Selection of seed cells

Regardless of the chosen design strategy of osteochondral scaffold, osteogenic and chondrogenic cells and biochemical factors could be pre-seeded concurrently and respectively to their corresponding phases. The cartilage layer of osteochondral scaffold was seeded with chondrogenic cells to generate cartilaginous construct, and the bone layer of osteochondral scaffold was seeded with osteogenic cells to generate bone-like construct [68]. In repair processes, cells migrated into defect areas and secreted ECM proteins resulting in neo-tissue formation. Therefore, the selection of an ideal cell source is significantly important to improve osteochondral repair efficiencies. The most important selection criterion for seed cells is the ability to produce tissue-specific ECM proteins and without risks of host immune responses and disease transmission. Secondary cell source should have no limitations in the amounts available and be easy to maintain desired phenotype *in vitro*.

Chondrocytes in adult articular cartilage account for only 1–5% of the total volume of hyaline cartilage (proximately 1.0 × 10^6^ cells/cm^3^ on average throughout the full thickness of mature cartilage) [52]. Cartilage is relatively a hypocellular tissue, but chondrocytes are essential since it is these cells that replace degraded matrix molecules to maintain the correct size and mechanical properties of the cartilage tissue. Chondrocytes could be seeded onto a scaffold and stimulated to produce a cartilage-like matrix in cartilage tissue engineering, so as to simulate the production of new cartilage tissue with the typical characteristics of native hyaline cartilage [121]. Autologous cells could avoid risks of immunological rejection and infectious diseases transmission [122]. However, the number of autologous chondrocytes from spare cartilage is limited because mature chondrocytes have a relatively low metabolic activity, which may hardly be adequate for the high demand of cells to constitute engineered cartilage. In addition, as the chondrocytes are cultured for longer periods before implantation, the cartilage formed is increasingly fibrous in nature [123].

Stem cell-based tissue engineering plays a significant role in skeletal system repair and regenerative therapies [124]. BMSCs are more plentiful, which could provide both osteogenic and chondrogenic cells while eliminating the risk of immunological rejection and infectious diseases transmission [122]. MSCs could be induced to form chondrocytes in chemically specified culture media supplemented with transforming growth factor-β (TGF-β) [125,126]. Under different culture conditions, MSCs could also be induced to form osteogenic cells, and both types of induction together may constitute a biphasic osteochondral construct graft from a single cell source [122]. It is proved that the BMSCs exhibit better chondrogenesis than MSCs of other origin, under presently defined culture and induction conditions [127]. Instead of chondrocytes, BMSCs could be expanded many-fold with little effect on the tissue that is eventually formed, making them prime candidates for transplantation in tissue-engineered constructs [126].

### 3.3 Choice of biochemical factors

Although the biomaterials consisting of osteochondral scaffolds are the foundations of the construct, they often require complementary biochemical factors that could improve tissue response, integration and repair. Generally, these biochemical factors consist of growth factors, gene delivery and small molecule-based drugs that could trigger appropriate response of endogenous cells after transplantation. The effects of biochemical stimuli on osteochondral therapy are shown in Table 2.

**Table 2:** The effects of the growth factor, gene delivery and small molecule as biochemical stimuli on osteochondral therapy.

Biochemical stimuli types		Effects of biochemical stimuli types on osteochondral unites	Ref
**Growth factor**	**TGF-β1**	Maintaining homeostasis of both articular cartilage and subchondral bone; Earlier modulator for cartilage repair before BMP-2 action with hyaline-like cartilage formation	[49,[144], [145], [146]]
	**FGF-2**	FGF-2 had a modulating effect on the defect-surrounding subchondral bone *via* upregulation of BMP-2, BMP-4 and SOX9 at the early stage; Low dose FGF-2 improved the repair upon directly injected to subchondral bone	[147]
	**SDF-1α**	Stimulate MSCs migration and homing	[148,149]
	**IGF-I**	Superior growth morphology and surface architecture of the neo-tissue; Increased chondrocyte viability	[150,151]
**Protein-coding gene**	**BMP-2**	*In vivo* BMP-2 causes osteochondral differentiation of MSCs even with short exposure; Combination of BMP-2 further enhanced osteochondral repair effects	[53,93,150]
	**SOX9**	Regulates the development and formation of cartilage	[134,135]
	**1L-1Rα**	IL-1Ra expression protected cartilage-derived matrix (CDM) hemispheres from inflammation-mediated degradation, and supported robust bone and cartilage tissue formation	[93]
**Small molecule**	**Dexamethasone**	A potent glucocorticoid with concomitant anti-catabolic and pro-anabolic effects on cartilage; Supporting the functional integrity of adjacent graft and host tissue while also attenuating inflammation caused	[137]
	**Berberine**	Exert significant immunosuppressive and anti-inflammatory effects; BER could upregulate the canonical Wnt signaling pathway to enhance the formation of subchondral bone	[138]
	**ALN**	Prevents bone resorption by inhibiting the activity of osteoclasts	[139]
	**KGN**	Induces chondrogenic differentiation of hBMSCs and inhibits catabolic reactions	[136]
	**BNTA**	Promotes generation of ECM components, suppressing inflammatory mediators	[142]
	**DIPQUO**	Markedly promotes osteoblast differentiation, and a significant increase in calcium matrix deposition	[143]
	**Y27632**	Promote the differentiation of chondroprogenitors; The effect on MSCs depends on cell density (low) and morphology (agglomerated)	[152]

Growth factors, a kind of cytokines that are secreted by many cell types, could either stimulate or prevent cellular adhesion, proliferation, differentiation, migration and gene expression, by up-regulating or down-regulating the synthesis of proteins, cytokines and receptors, influencing development, remodeling and repair of tissue [128]. It has been proved that cartilage growth and maturation were supported by growth factors, including TGF-β1, insulin-like growth factor-1 (IGF-1), fibroblast growth factor-2 (FGF-2) and bone morphogenetic proteins (BMP-2) [129]. Like cartilage, bone also possesses a plethora of growth factors, including BMPs, IGF-1/2, TGF-β and FGFs [130]. It is well known that the complex healing process in osteochondral defect is rely on the combined action of numerous signaling molecules which play distinct specific roles at different stages of osteochondral lesion repair. To provide therapeutic dosages in an appropriate time frame for the promotion of osteochondral tissue remodeling, it is requirement for control growth factor release and differential release profiles with tight temporal and spatial control. Therefore, further optimization is needed to achieve an adjustable and reproducible growth factor delivery system that could trigger cartilage and bone repair mechanisms [131].

Gene therapy might represent a promising strategy for osteochondral defects repair through transfecting cells to enhance the sustained expression of the protein of interest or through silencing target genes associated with bone and joint disease, which lead to more effective site-specific and prolonged effects [132]. Scaffold-based gene delivery not only provides more adjustable release with temporal control, but also allows for spatial distribution of osteogenic and chondrogenic genes, which helps achieve zonal differentiation of progenitor cells and well-defined bone and cartilage layers [133]. The most commonly used genes in osteochondral gene therapy include encoding for growth factors like BMP-2 and TGF-β3 [53,93,134], encoding for anti-inflammatory molecules, such as interleukin-1 receptor antagonist (IL-1RA) [93], and encoding for transcription factors like SOX9 [134,135]. However, many gene delivery approaches rely on viral vectors, which improve transfection efficiency and thus gene expression levels, but are also related to the risk of immune recognition, response and neutralization [132]. In addition, difficulties in achieving permanent transgenic expression and producing targeted proteins at optimal concentrations also limit the effectiveness of gene therapy in osteochondral disease treatment.

Small molecule drugs could also be common and effective cell-instructive factors in tissue engineering due to its easy high-throughput screening, simple administration and low cost. The effect of small molecule is normally dose-dependent allowing for a fine-tuning of their biological action [124,136]. Therefore, many studies have focused on the identification and synthesis of small molecule drugs that could induce osteogenesis and chondrogenesis for potential osteochondral defect treatment. Dexamethasone is a potent glucocorticoid with concomitant anti-catabolic and pro-anabolic effects on cartilage and could be serve as an adjunct for osteochondral repair strategies [137]. Berberine, a plant alkaloid, has osteoinductive properties and is capable of promoting osteochondral regeneration *in vivo*, combined with an interpenetrating network scaffold of sodium hyaluronate and sodium alginate [138]. Alendronate (ALN) could promote osteogenesis of the MSCs [139]. Kartogenin (KGN) induces chondrogenic differentiation of hBMSCs [136] and inhibits catabolic reactions by up-regulating tissue inhibitors metalloproteinases (TIMPs) expression and decreasing matrix metalloproteinases (MMPs) expression [140,141]. N-[2-bromo-4-(phenylsulfonyl)-3-thienyl]-2-chlorobenzamide (BNTA) could stimulate cartilage ECM production and exert a protective and regenerative effect in osteochondral defect model, by upregulating gene and protein expression of superoxide dismutase 3 (SOD3) [142]. 6,8-dimethyl-3-(4-phenyl-1H-imidazole-5-yl) quinolin-2(1H)-one (DIPQUO), is another novel small molecule proven to induce osteogenic differentiation of hMSCs and stimulate bone mineralization [143]. 1R,4r)-4-((R)-1-aminoethyl)-N-(pyridin-4-yl) cyclohexane-carboxamide (Y27632), could increase the differentiation of chondroprogenitors, but its.

Effect on MSCs depends on cell density and morphology [153,154]. A significant limitation of small molecule therapy is its lower target specificity compared to protein agents, which may cause deleterious side effects.

As with any other therapeutic candidate, biochemical factors could be directly loaded during or after scaffold fabrication, which have realistically been applied in osteochondral tissue and identified as clinically important roles in tissue regeneration. To design and develop an optimum system so that the right signals might be transmitted for both kinds of tissue, some factors are crucial, such as extensive safety screenings, the controlled delivery and high target specificity of biochemical factors [155].

## 4 Construct techniques of IGTEOS *in vitro*

Various techniques have been developed to prepare IGTEOS, such as sequential layering of slurry or hydrogel solutions at partial gelation, 3D printing, electrospinning, microfluidic-based method, buoyancy-driven approach, magnetic field control and buoyancy-driven approach. In general, the strategies for fabricating IGTEOS refer to the deposition of materials at different spatial coordinates along the gradient axis, which could be roughly summarized as additive manufacturing. Chemical compositional gradients involve the changes in the fundamental materials and the encapsulated bioactive molecules. In addition, stratifications in the content of minerals, and porosity and pore size are common approaches for gradient osteochondral scaffold. To provide comprehensive overview of fabrication techniques for IGTEOS, we have summarized key techniques for fabricating IGTEOS *in vitro*, explored their advantage and limitations [36], and outlined different gradients, such as compositional, architectural and mechanical properties, as shown in Table 3. Moreover, we have summarized the most recent studies about gradient tissue-engineered osteochondral scaffolds by additive manufacturing strategies in Table 4.

**Table 3:** Gradient fabrication strategies and their key methods, respective advantages and limitations.

Strategies	Key methods	Advantage	Limitations	Established gradient	Ref
**Additive manufacturing**	**Sequential layering**	Rapid and simple protocol No specialist equipment	Restricted to stepped transitions Risk of delamination	Architectural	[29,61,68,157]
				Compositional	[7,29,59,68,81,87,157,158]
				Mechanical	[61,158]
	**Electrospinning**	Rapid and simple protocol Can form continuous gradients Can form a range of gradients	Restricted to thin scaffolds Challenging with live cells	Architectural	[159,160]
				Compositional	[159,160]
				Mechanical	[159]
	**3D printing**	Free-form control over the material architecture Can form continuous gradients Can form a range of gradients	Requires printable materials Requires specialist equipment and significant user expertise	Architectural	[73,79]
				Compositional	[73,161]
				Mechanical	[42,73,78]
	**Fluid mixing**	Rapid and simple protocol Can form continuous gradients Can form a range of gradients	Restricted to single gradients	Architectural	[162,163]
				Compositional	[162,164,165]
				Mechanical	[162,164]
**Other technique**	**Buoyancy**	Can form continuous gradients Rapid and simple protocol	Requires a density difference	Architectural	[116,118]
	**Magnetic fields**		Requires magnetic particles	Compositional	[[116], [117], [118]]
	**Electric field**		Requires field responsivity	Mechanical	[118]

**Table 4:** Summary on the most recent gradient osteochondral scaffolds by additive manufacturing strategies.

Scaffold composition	Fabrication technique	Established gradients	Main finding	Ref
Top: PolyHEMA/HAc Bottom: PolyHEMA/nHA	Sphere-templating technique	Composition	The integrated bi-layered scaffold could support simultaneous matrix deposition and adequate cell growth of two distinct cell lineages in each layer during four weeks of co-culture *in vitro*	[29]
		Porosity		
		Stiffness		
Top: SF Medium: SF/nHA Bottom: SF/nHA	Paraffin sphere leaching and modified temperature gradient-guided TIPS technique	Composition Porosity Stiffness	A chondral layer with a longitudinally oriented microtubular structure, a bony layer with a 3D porous structure and an intermediate layer with a dense structure. The trilayered and integrated osteochondral scaffolds could effectively support cartilage and bone tissue generation *in vitro*	[68]
Top: Col-I/Col-II/HAc (5/15/2) Med: Col-I/Col-II/HA (5/5/2) Bottom: Col-I/HA (1/2)	Iterative layering freeze-drying	Composition Porosity Stiffness	The multi-layered scaffold had a seamlessly integrated layer structure, homogeneous cellular distribution throughout the entire construct. **Rabbits model:** tissue regeneration with a zonal organization	[54,166]
Top: Col-II/(CaP/pTGF-β3/CaP/PEI nanoparticles) Bottom: Col-I/nHA/(CaP/pBMP −2/CaP/PEI nanoparticles)	3D enzymatic-crosslinked gene-activated	Composition Porosity Stiffness	The sustained release of incorporated plasmids from bilayer scaffolds promoted long-term transgene expression to stimulate hMSCs differentiation into the osteogenic and chondrogenic lineages by spatial and temporal control, which accelerate healing process	[87]
Top: Silicified silk/R5 (1/62.5) Medium: Silicified silk/R5(1/125) Bottom: silicified silk/R5(1/250)	Sequential laying and then crosslinked	Composition	The gradient silicified silk/R5 composites offers continuous transitions in cytocompatibility and biodegradability, and promoted and regulated osteogenic differentiation of hMSC in an osteoinductive environment	[58]
		Porosity		
		Stiffness		
Top: CS/HAc Bottom: CS/SA/HA	Thermally-induced phase separation (TIPS)	Composition	Cell proliferation and migration to the interface along with increased gene expression associated with relevant markers of osteogenesis and chondrogenesis	[158]
		Porosity		
		Stiffness		
Top: PGA/Ly/SA/BC/mHA Bottom: PGA/Ly/SA/BC/nHA	Three-step crosslinking procedure	Porosity Stiffness	**Rabbits model:** good integration between the neo-subchondral bone and the surrounding host bone and the same thickness between the neo-cartilage and the surrounding normal cartilage	[61]
Top: GelMA-PDA/TGF-β3 Bottom: GelMA-PDA/HA/BMP-2	Simultaneously polymerizing layers using one-pot method	Composition	PDA fix and release proteins or growth factors, which endows the hydrogel with good cartilage and subchondral bone regeneration abilities.	[81]
		Porosity		
		Stiffness		
Top: Col-I	Sequentially stacked, crosslinked, and collectively lyophilize	Composition	**Rat model:** subcutaneous implantation in rats showing the gradient scaffold was significantly colonised by host cells and minimal foreign body reaction, confirmed its *in vivo* biocompatibility	[55]
Medium: HA/Col-I (10/90 and 30/70)		Porosity		
Bottom: HA/Col-I (1/1)		Stiffness		
Top: NC/PdBT Bottom: GHK/PdBT	Click conjugation of developmentally inspired peptides	Composition	**Rabbits model:** presentation of the NC peptide and incorporation of MSCs throughout the entire construct enhanced subchondral bone filling and the degree of bone bonding with adjacent tissue	[59]
Top: PEGDA Bottom: low-molecular-weight gels (LMWGs)	Assembly/disassembly of LMWGs inside the network by photopolymerization	Composition Porosity Stiffness	Each domain had an individual capacity to spatially control the differentiation of MSCs toward osteoblastic lineage and chondrocytic lineage. **Rabbits model:** the multi-domain gels distinctly improved the regeneration of subchondral bone and cartilage tissues	[85]
Top: ChS-NPs/SA/PVA Bottom: n-HA/SA/PVA	Injectable semi-interpenetrating	Composition	**Rabbits model:** the engineered osteochondral mimetic injectable hydrogel with spatial variation, deep mineralized zone and gradient interface showed accelerated osteochondral tissue regeneration	[62]
		Porosity		
		Stiffness		
Top: TGF-β1/PLGA NPs	Table-top stereolithography 3D printing	Composition Porosity	Scaffolds with a highly interconnected microporous calcified transitional and subchondral region were created which facilitated cell adhesion, proliferation, and cellular activities	[167]
Medium: 10%nHA				
Bottom: 20%nHA				
Top: GelMA-PEGDA/TGF-β1-PLGA NPs	3D stereolithography printing	Composition Stiffness	Scaffold promoted osteogenic and chondrogenic differentiation of hMSCs, as well as enhanced gene expression associated with both osteogenesis and chondrogenesis alike	[43]
Bottom: GelMA-PEGDA/nHA				
Top: PCL				
Bottom: PCL/HA	Selective laser sintering technique	Composition Stiffness	**Rabbit model:** Scaffolds induced cartilage formation by accelerating the early subchondral bone regeneration, and the newly formed tissues could well integrate with the native tissues	[7]
Top: PNAGA-PTHMMA/TGF-β1 Bottom:PNAGA-PTHMMA/β-TCP	Thermal-assisted extrusion printing	Composition	**Rat model:** 3D-printed biohybrid gradient hydrogel scaffolds significantly accelerate simultaneous regeneration of cartilage and subchondral bone	[42]
		Porosity		
		Stiffness		
Top: PACG-GelMA/Mn^2+^ Bottom: PACG-GelMA/BG	Low-temperature receiver assisted 3D-Printing	Composition Stiffness	Scaffold enhances gene expression of chondrogenic-related and osteogenic-related differentiation of hBMSC. **Rat model:** significantly facilitates concurrent regeneration of cartilage and subchondral bone	[86]
Top: PCL/PDA/TGF-β1 Bottom: PCL/nHA	Fused deposition modeling 3D printing and casting	Composition	3D printed constructs with nHA and bioactive cues have improved mechanical properties and enhanced hMSC adhesion, growth, and differentiation	[72]
		Porosity		
		Stiffness		
Top: Peptide/TCP/PLGA Bottom: P(DLLA-TMC)/Col-I	Cryogenic 3D printing	Composition	High viability and proliferation at both subchondral-and cartilage layer. Moreover, gradient rBMSC osteogenic/chondrogenic differentiation was obtained in the osteochondral scaffolds	[78]
		Porosity		
		Stiffness		
Top: PCL Bottom: PCL/nHA	Multi-material extrusion 3D printing	Composition	The fabricated scaffolds incorporate porosity changes similar to those found in the native osteochondral unit as well as compressive properties in the range of human trabecular bone	[73]
		Porosity		
		Stiffness		
Top: PCL	Multi-nozzle 3D printer	Composition	More cells attached and grew vigorously on the sintered HA layers and PCL layers, and proliferated very fast with days	[71]
Bottom: HA		Stiffness		
Top: HAc/KGN hydrogel Bottom: HA/ALN	3D-printing and semi-immersion	Composition	**Rat model:** Scaffold had sufficient anchoring strength to maintain stable binding of the two layers, and strong promotions of cartilage or bone regeneration in the respective layers	[139]
		Porosity		
		Drug-factor		
Top: fibrin Bottom: CS-Mg8	Porogen-leaching method and 3D printing	Composition	**Rabbit model:** the biphasic scaffold could achieve simultaneous regeneration of cartilage and subchondral bone, the neo-tissue was well connected to the host tissue, and the tidemark was obvious in the neo-tissue	[168]
		Porosity		
		Stiffness		

### 4.1 Sequential laying

Early additive manufacturing methods used adhesives to bind two or more solid biomaterial layers. Alginate-boronic acid glues, such as agarose, acrylamide, and chitosan-catechol, have been developed to bind precast hydrogels [156]. However, the attractive interactions within each material layer are generally stronger than those bridging the interface, which might result in delamination between the stacked layers. The addition of a liquid precursor to a mold followed by partial crosslinking could be repeated to build sequentially layered structures, which could generate material layers without requiring an intermediary adhesive.

**Fig. 6:**
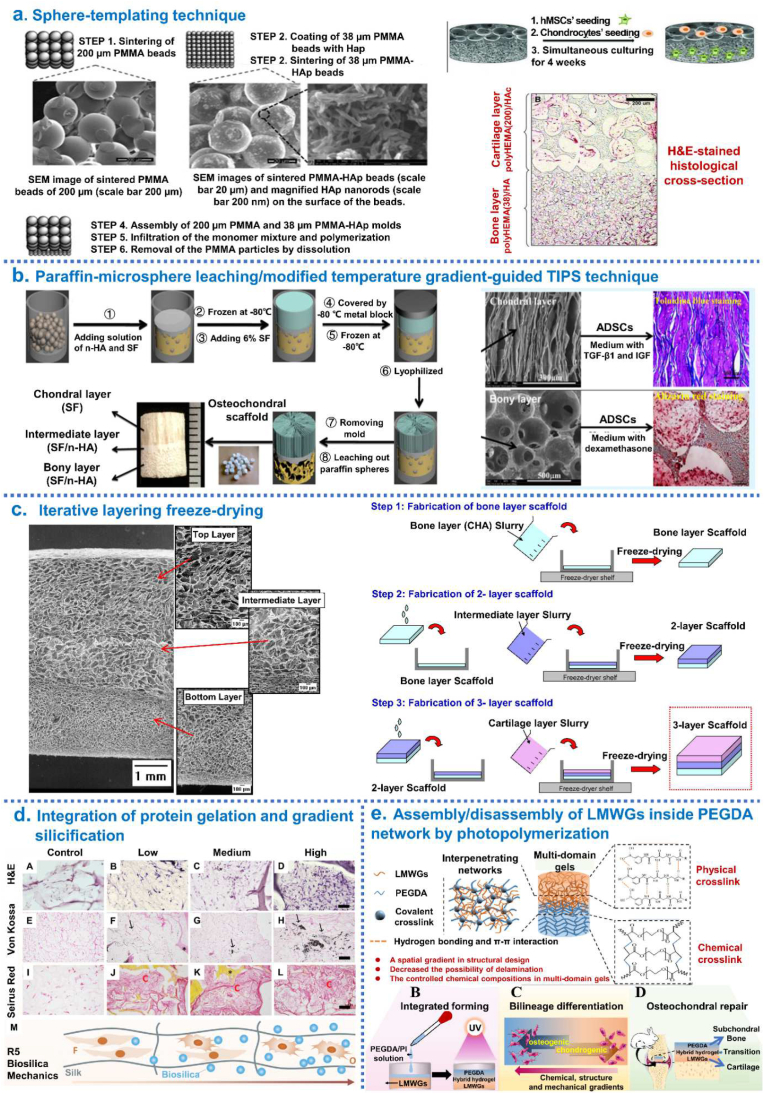
Integrated hierarchical osteochondral scaffold was designed by sequential layering techniques. (a) Steps of the sphere-templating technique to fabricate an integrated bi-layered scaffold and *in vitro* cell study. (b) Schematic diagrams of the process for preparing integrated osteochondral scaffolds by combining paraffin-sphere leaching with a modified temperature gradient-guided thermal-induced phase separation (TIPS) technique. (c) The “iterative layering freeze-drying” fabrication process diagram to fabricate collagen-based scaffold with a seamlessly integrated layer structure for osteochondral defect repair. (d) The process of generate seamlessly integrated bilayer hydrogel for osteochondral defect repair by simultaneously polymerizing two layers using a one-pot method. (e) The mechanism of formation of the multi-domain gel and its great potential for osteochondral regeneration through controlling chemical, structural, and mechanical properties of each gel domain. Reproduced with permission: (a) [29], copyright 2013, Wiley; (b) [68], copyright 2014, ACS; (c) [54], copyright 2014, Elsevier; (d) [81], copyright 2019, Wiley; (e) [85], copyright 2021, Elsevier.

Galperin et al. [29] reported the design and fabrication of an integrated bi-layered scaffold based on a degradable poly (2-hydroxyethyl methacrylate) (polyHEMA) hydrogel by sphere-templating technique. Specifically, the cartilage layer of the scaffold was composed of degradable polyHEMA with covalently incorporated hyaluronic acid (HAc) and had pore diameters of 200 μm, while the bone layer was decorated with nHA and had pore diameters of 38 μm. This bi-layered scaffold could support simultaneous matrix deposition and adequate cell growth of two distinct cell lineages in each layer (Fig. 6a). Ding et al. [68] fabricated a biomimetic integrated tri-layered osteochondral scaffold consisting of silk fibroin (SF)/HA by combining paraffin-microsphere leaching with the modified temperature gradient-guided thermally-induced phase separation (TIPS) technique, which could effectively support cartilage and bone tissue generation *in vitro* (Fig. 6b). Levingstone et al. [54,166] reported an “iterative layering freeze-drying” approach using multiple steps of freeze-drying, crosslinking and rehydration to create tri-layered osteochondral scaffolds. This novel scaffold mimicked the inherent gradient structure of healthy osteochondral tissue achieving a seamlessly integrated layer structure. The *in vivo* studies indicated that osteochondral tissue regeneration with a zonal organization in rabbit model (Fig. 6c). Lee et al. [87] developed 3D enzymatic-crosslinked gene-activated bilayer Col-II/Col-I-nHA scaffold containing CaP/pDNA/CaP/PEI nanoparticles with encapsulated TGF-β3 and BMP-2, respectively. MSCs were induced by plasmid TGF-β3 and plasmid BMP-2 in different layers to simultaneously support the regeneration of articular cartilage and subchondral bone. Guo et al. [58] designed biomimetic gradient silicified silk/R5 (GSSR5) composites through integration of enzymatically triggered protein gelation and R5-induced gradient silicification, which presented a continuous transition in terms of composition, structure and mechanical properties as well as cytocompatibility and biodegradability. The *in vitro* results demonstrated that cell differentiation along the GSSR5 composites. Inspired by mussel chemistry, Gan et al. [81] developed a seamlessly integrated bilayer hydrogel for osteochondral defect repair by simultaneously polymerizing two layers using a one-pot method: self-polymerized dopamine (PDA) noncovalent interactions with gelatin methacrylamide-polydopamine (GelMA) to form GelMA-PDA hydrogel including TGF-β3 acted as a cartilage layer; *in situ* mineralized HA homogeneously dispersed throughout the GelMA-PDA hydrogel to form the GelMA-PDA/HA hydrogel including BMP-2 acted as a subchondral bone repair layer; the pre-polymerization solution for GelMA-PDA was poured into GelMA-PDA/HA hydrogels in a mold, and then upper and lower layers were gelled simultaneously by thermal-initiated polymerization to generate seamlessly integrated bilayer hydrogel. Such hydrogel with good cartilage and subchondral bone regeneration abilities (Fig. 6d). In 2020, Parisi et al. created integrated osteochondral scaffolds, that was, the slurries of Col-I and HA at varying ratios sequentially stacked, crosslinked and collectively lyophilized to form scaffolds, exhibiting good biological performances both *in vitro* and *in vivo* [55]. In 2021, Guo et al. [59] have developed a bi-layered and tissue-specific hydrogel system by the click conjugation of developmentally inspired peptides (a chondrogenic N-cadherin peptide (NC) and an osteogenic glycine-histidine-lysine peptide (GHK)) to stratified hydrogel layers. In this system, the crosslinker poly (glycolic acid)-poly (ethylene glycol)-poly (glycolic acid)-di (but-2-yne-1,4-dithiol) (PdBT) was click conjugated with either a cartilage- or bone-specific peptide sequence of interest, and then mixed with a suspension of thermo-responsive polymer and MSCs to generate tissue-specific, cell-encapsulated hydrogel layers targeting the cartilage or bone. Through the assembly/disassembly of low-molecular-weight gels (LMWGs) inside the stable poly (ethylene glycol) diacrylate (PEGDA) network by photopolymerization, Zhang et al. [85] developed a multi-domain gel with chondrogenic-osteogenic gradient transition. The *in vitro* studies demonstrated that each domain had an individual capacity to spatially control the differentiation of MSCs toward osteoblastic lineage and chondrocytic lineage (Fig. 6e). In addition, sequential laying was highly compatible with other fabrication methods. Radhakrishnan et al. [62] have developed an injectable semi-interpenetrating network hydrogel construct with chondroitin sulfate nanoparticles (ChS-NPs) and nHA (30–90 nm) in chondral and subchondral hydrogel zone, respectively. SEM of *in situ* formed hydrogel longitudinal sections of cartilage, subchondral and interfacial regions exhibiting gradient microstructure.

Stacking layers method is a rapid and simple approach that does not require specialist equipment, but the stepped transition results in a material consisting of discrete layers rather than a continuous gradient. Continuous transitions possess greater relevance to most natural systems, enabling improved load transmission and avoiding interfaces that could present mechanical instability or exclude cells.

### 4.2 Electrospinning

Generally, electrospun structures are characterized by high surface-to-volume ratio and high porosity, showing morphological similarities to the natural ECM [[169], [170], [171]]. Electrospinning could also be used to prepare materials with gradients in morphological and mechanical properties by controlling the fluid deposition process, that is, reservoirs loaded with different polymer solutions are sequentially deposited onto a moving collector by electrospinning technique. Bidirectional gradient electrospinning provides an alternative to simple material stacking to create gradient scaffolds, that is, the two solutions are simultaneously electrospun onto the collector at an inversely proportional flow rate. Electrospinning could produce gradient materials in numerous ways and is highly compatible with other fabrication methods. In particular, the integration of electrospun membranes into microfluidic chips, enabling accurate and tunable mixing of the precursor solutions with variable nanoparticles and biomolecule concentrations before the electrospinning process, could produce nanofibers with spatially controlled gradients and enhanced functionality.

Erisken et al. [172] prepared a PCL mesh with controlled gradation of insulin and β-glycerophosphate (β-GP) concentrations in between the two sides of a nanofibrous scaffold, which achieved *via* the application of the twin-screw extrusion and electrospinning method. Chondrogenic differentiation of the human adipose-derived stromal cells increased at insulin-rich locations and mineralization increased at β-GP-rich locations. Zhang et al. [173] produced gradient electrospinning nanofibers by using a two inlets microfluidic device in combination with an electrospinning nozzle on a 3-D controllable platform, which could guide the spatial differentiation of MSCs. Mohan et al. [174] fabricated the fiber-hydrogel hybrid scaffolds by layer-by-layer arrangements of electrospun PCL fiber mats (containing a dual gradient of chondroitin sulfate and bioactive glass) within an agarose-gelatin hydrogel to mimic the native osteochondral interface. Zhao et al. [175] developed a strategy for incorporating cellulose acetate (CA) in emulsion electrospun bFGF-containing PLGA scaffolds *via* dual-source dual-power electrospinning (DSDP-ES) technology. In this process, either bilayer scaffolds or trilayer scaffolds were made through sequentially conducting CA electrospinning and bFGF-containing PLGA emulsion electrospinning, which could achieve an enhanced, steady and sustained release of bFGF, and be beneficial to tissue regeneration. Liu et al. [159] developed a self-developed 3D bioprinting platform combining extrusion deposition with multi-nozzle electrospinning to fabricate the functional gradient scaffold with multidrug spatiotemporal release profiles. Qu et al. [83] develop a tricomponent scaffold consisting of rapidly degrading poly (ethylene oxide) (PEO) with collagenase, slower-degrading HA with platelet-derived growth factor-AB (PDGF-AB), and PCL to enable direct cell migration for connective tissue repair, which sequentially release active collagenase (to increase ECM porosity) and PDGF-AB (to attract endogenous cells) in a localized and coordinated manner.

Overall, electrospinning technology is simple, robust and cost-effective, but its application is limited due to unidirectional gradient, poor control over scaffold architecture and external environment (e.g., temperature and humidity). Moreover, the electrospinning process is damaging to cells due to the use of cytotoxic solvents and possible shear forces upon extrusion.

### 4.3 Controlled fluidic mixing

In recent years, a new equipment for preparing gradient materials by continuous deposition have been developed. The “gradient maker” could produce different gradients by controlling the relative flow rates during the casting process, in which continually feed solutions from different reservoirs into a single joined outlet and then the mixed liquid deposited and cast in a mold.

Zhu et al. [164] generated mechanically graded cartilage tissue constructs by using gradient maker consists of interconnected vertical chambers which filled with the chondrocyte-laden hydrogel precursor solution with two different biopolymer concentrations. Such gradient hydrogel, composed of 8arm-PEG-norbornene, PEG-dithiol and 25% methacrylated chondroitin sulfate (CSMA), could provide a 3D artificial cell niche to enable tissue engineering of various tissue types with zonal organizations or tissue interfaces (Fig. 8a). Hubka et al. [176] designed a versatile multichannel gradient maker device (MGMD) to create desired gradients of perlecan domain I across HAc-based hydrogels. This study concluded that establishing covalently-bound perlecan domain I (PlnD1) gradients in hydrogels provided a new means to establish physiologically-relevant gradients of Heparin-binding growth factor (HBGF) that were useful for a variety of applications in tissue engineering (Fig. 8b). Using on-demand reconfigurable microfluidics, Costantini et al. [163] fabricated gelatin/HA scaffolds with gradients pore size: gelatin solution was loaded with nHA particles, foamed using the valve-based flow-focusing chip to synthesize graded materials, and then crosslinked, lyophilized and sintered the samples. The presented technology opened new possibilities in microporous material synthesis. By combination of microfluidics with extrusion-based bioprinting and instructive bioinks, Idaszek et al. [162] mixed doped alginate-based solutions for the preparing of graded cell-laden constructs to mimic the ECM organization of native cartilage. This technique facilitated the deposition of continuous gradients of chemical, mechanical and biological cues and fabrication of scaffolds with very high shape fidelity and cell viability (Fig. 8c). Xin et al. [165] developed a microfluidic method combining a micro-fluidic mixer module and a droplet generator module to generate gradient PEG-based Microporous annealed particle (MAP) hydrogel scaffolds. Specifically, microgels with varying properties were produced by adjusting the polymer components and the relative flow rates between two precursor solutions, collected layer-by-layer in a syringe and then annealed with thiol-ene click chemistry to form hydrogel scaffold with continuous physicochemical gradient (Fig. 8d). This method of generating spatial gradients in MAP hydrogels could be further used to study cell-material interactions.

**Fig. 7:**
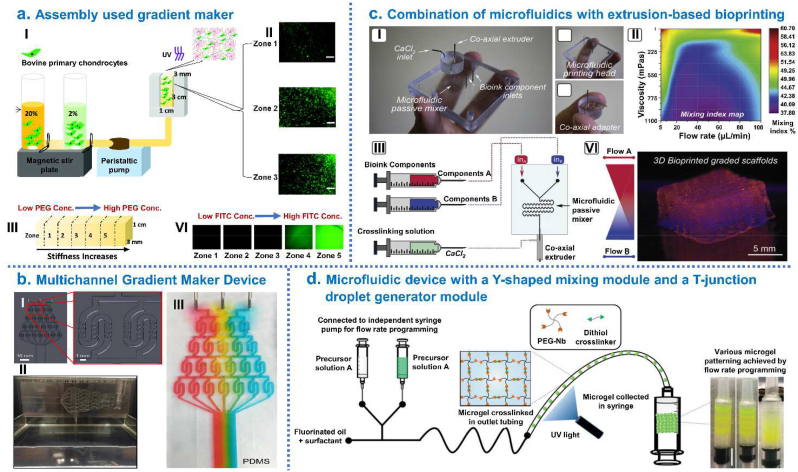
Integrated hierarchical osteochondral scaffold was designed by 3D printing techniques. (a) Preparation of biphasic scaffold by 3D stereolithography printer: GelMA-PEGDA as primary ink, TGF-β1/PLGA NPs loaded into the top layer and nHA loaded into the bottom layer of osteochondral scaffold. (b) Fabrication of a bio-inspired multilayer osteochondral scaffold that consisted of the PCL and HA/PCL microspheres via selective laser sintering layer-by-layer process. (c) Fabrication of biohybrid gradient PNT scaffolds by thermal-assisted extrusion 3D printing for repair of osteochondral defect. (d) 3D printing gradient PACG-GelMA hydrogel scaffolds assisted with a low-temperature receiver: the bioactive Mn^2+^ are loaded into the top cartilage layer while the BG is incorporated into the bottom subchondral bone layer. (e) Fabrication process of tissue-engineered osteochondral scaffolds through integrate fused deposition modeling 3D printing with a casting technique. (f) Fabrication of biphasic HA/PCL scaffolds by multi-nozzle 3D printer. Reproduced with permission: (a) [43], copyright 2019, Elsevier; (b) [7], copyright 2017, Elsevier; (c) [42], copyright 2018, Wiley; (d) [86], copyright 2019, Wiley; (e) [72], copyright 2019, Elsevier; (f) [71], copyright 2021, Springer.

**Fig. 8:**
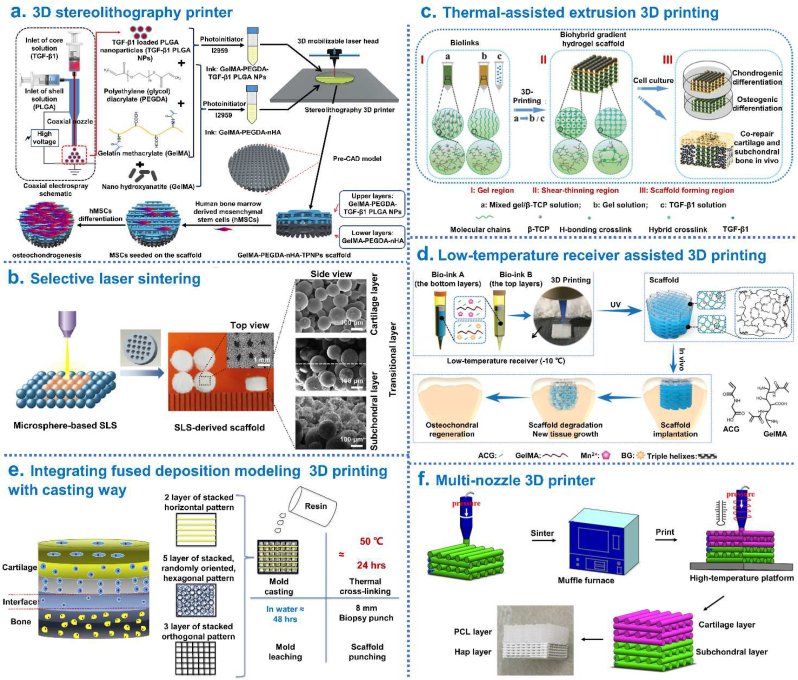
Integrated hierarchical osteochondral scaffold was designed by controlled fluidic mixing techniques. (a) Gradient hydrogel fabrication and characterization: (I) Schematic representation of gradient maker assembly used to make gradient hydrogel which is bulk polymerized after the prepolymer solution is mixed with bovine primary chondrocytes; (II) Cell viability within selected zones of the gradient hydrogel on day; (III) Compressive modulus from zone 1 to zone 5 in gradient hydrogel; (VI) Dual-gradient hydrogel with biochemical model protein (FITC tagged Bovine Serum Albumin-BSA) encapsulation could also be achieved. (b) Development of Multichannel Gradient Maker Device (MGMD): (I) Solidworks 3D computer-aided design software used to design the MGMD to facilitate chaotic mixing in channels; (II) PDMS MGMDs were generated using 3D printed molds and a syringe pump was used to flow solutions through MGMD channels; (III) Colored dyes were mixed with 70% glycerol and pumped through the MGMD to visually display gradient generation. (c) Combination of microfluidics with extrusion-based bioprinting and instructive bioinks to produce graded scaffolds: (I) Microfluidic extrusion system composed of the microfluidic printing head and the co-axial adapter; (II) Mixing index heatmap; (III) schematically shown how to 3D bioprint graded scaffolds. (d) Microgel production procedure using a microfluidic device with a Y-shaped mixing module and a T-junction droplet generator module. Right side photograph showing examples of microgel patterning. Reproduced with permission: (a) [164], copyright 2018, Mary Ann Liebert; (b) [176], copyright 2019, Elsevier; (c) [162], copyright 2019, IOP; (d) [165], copyright 2020, Wiley.

Controlled fluidic mixing could produce tissue-engineered scaffold with continuous gradients by controlling the relative flow rates during the casting process. However, similar to the previously mentioned techniques, the above fabrication techniques for IGTEOS do not provide precise control over pore size, micro-structure and pore interconnectivity.

### 4.4 3D printing

The emerging 3D printing techniques could achieve structural complexity of scaffolds for precise and personalized therapy of osteochondral defect, which employ layer-by-layer deposition and computer-aided design (CAD) for scaffold production. The most common methods are selective laser sintering, fused deposition modelling, stereolithography, inkjet 3D printing and extrusion-based 3D printing [177].

With the use of CAD software and table-top stereolithography 3D printer, Castro et al. [167] fabricated a porous and highly interconnected osteochondral scaffold by table-top stereolithography 3D printing, which contained a gradient of nHA within the highly porous subchondral bone layer and chondrogenic TGF-β1 nanospheres in the cartilage layer for enhanced osteochondral regeneration. This work served to illustrate the efficacy of the nano-ink and current 3D printing technology for efficient fabrication of the osteochondral scaffold. A similar approach was described by Zhou and coworkers in 2019 to fabricate biomimetic osteochondral scaffolds for well osteochondral repair and regeneration [43]: TGF-β1 encapsulated core-shell nanoparticles (TGF-β1/PLGA NPs) were prepared *via* a co-axial electrospraying method; GelMA and polyethylene (glycol) diacrylate (PEGDA) were utilized for the preparation of the primary ink (GelMA-PEGDA), and then nHA and TGF-β1/PLGA NPs were distributed separately into the lower and upper layers. The finding demonstrated that 3D printed biphasic structure were excellent candidates for osteochondral repair and regeneration (Fig. 7a). Du et al. [7] constructed a bio-inspired multilayer osteochondral scaffold that consisted of the polycaprolactone (PCL) and HA/PCL microspheres *via* selective laser sintering layer-by-layer process. The precisely-designed multilayer scaffold featured a macro-porous cylinder with a continuous HA gradient from the articular cartilage layer to the subchondral bone layer. The *in vivo* study demonstrated that scaffolds induced cartilage formation by accelerating the early subchondral bone regeneration (Fig. 7b). Gao et al. [42] synthesized a high-strength thermo-responsive supramolecular copolymer hydrogel (PNT) by one-step copolymerization of dual hydrogen bonding monomers: N-acryloyl glycinamide (NAGA) and N-[tris(hydroxymethyl)methyl] acrylamide (THMMA). The biohybrid gradient PNT hydrogel scaffolds with precisely loaded TGF-β1 on the top layers and β-TCP particles on the bottom layers were prepared by thermal-assisted extrusion printing using a device equipped with three cartridges which were controlled to perform alternate printing by the predesigned program. The *in vivo* experiments revealed that the 3D-printed scaffolds significantly accelerate simultaneous regeneration of cartilage and subchondral bone in a rat model (Fig. 7c). Furthermore, using 3D-bioprinting method of the biohybrid gradient scaffolds assisted with a low-temperature receiver, Gao et al. [86] fabricated high-strength biohybrid gradient scaffold consisting of top layer of cleavable poly(N-acryloyl 2-glycine) (PACG) and methacrylated gelatin (GelMA) hydrogel-Mn^2+^ (PACG-GelMA/Mn^2+^) and bottom layer of PACG-GelMA hydrogel-bioactive glass (PACG-GelMA/BG) for osteochondral defects repair. Around 12 weeks after *in vivo* implantation, the hydrogel scaffold significantly facilitated concurrent regeneration of cartilage and subchondral bone in a rat model (Fig. 7d). By integrating fused deposition modeling (FDM) 3D printing with a casting technique, Nowicki et al. [72] fabricated multiphasic osteochondral construct with different layer geometries: the PCL based shape memory material was used as the osteochondral matrix material, nHA was printed into the subchondral bone layers and chondrogenic growth factors were fabricated into the cartilage layer, to achieve a spatially appropriate osteogenic and chondrogenic response (Fig. 7e). To obtain integrated tissue-engineered osteochondral scaffolds which were structurally and mechanically similar to native osteochondral tissue, wang et al. [78] produced closely bonded subchondral layer (peptide/TCP/PLGA) and cartilage frame (thermal-responsive poly (D, l-lactic acid-co-trimethylene carbonate) (P(DLLA-TMC))) through cryogenic 3D printing, and further dispensing of TGF-β1/collagen I hydrogel into the cartilage frame. This study provided a facile way to produce integrated osteochondral scaffolds for concurrently directing rBMSC osteogenic/chondrogenic differentiation at different regions. In 2019, Bittner et al. [73] described the fabrication of porous PCL and PCL-nHA scaffolds with incorporated vertical porosity and ceramic content gradients *via* a multi-material extrusion 3D printing system for osteochondral tissue engineering, which could better address the simultaneous gradients in architecture and mineralization found in native osteochondral tissue. In 2021, Suo et al. [71] designed a novel biphasic scaffold with HA and PCL using a multi-nozzle 3D printer. This biphasic HA/PCL scaffold could take advantage of both the rigidity of HA and the elasticity of PCL, thus had biomimetic mechanical properties for its further applications (Fig. 7f).

In addition, 3D printing could be combined with other manufacturing methods to produce gradient materials for osteochondral defect repair. Liu et al. [139] developed a semi-embedded biomimetic biphasic osteochondral scaffold with the layer-specific release of stem cell differentiation inducers. Specifically, the HAc hydrogel was employed as the cartilage-regeneration layer, which was mechanically enhanced by host-guest supramolecular units to control the release of kartogenin (KGN). The bone-regeneration layer was a 3D-printed HA scaffold releasing alendronate (ALN). The two layers were bound by semi-immersion and could regulate the hierarchical targeted differentiation behavior of the stem cells. In 2018, Shen et al. [168] reported a biphasic scaffold integrated by macro-porous fibrin and 3D-printed wollastonite scaffolds for osteochondral defect repair. *In vivo* transplantation of the biphasic scaffolds could induce the regeneration of both cartilage and subchondral bone to a great extent.

The main benefit of 3D printing is the precise control over scaffold architecture, which enables the generation of gradient construct that perfectly fits the lesion, paving the way for personalized therapy. Complex compositional, mechanical and structural gradients could be produced resembling the osteochondral tissue by tuning the material/hydrogel composition, construct architecture and encapsulated cell types during processing and subsequent polymerization. Nevertheless, the mechanical properties of the constructed structures by 3D printing are usually too poor to transplant and cultivate, causing them to be impractical for clinical use. And the cell behavior of the constructed objects is also a key factor determining the function of the construct. In addition, 3D printing requires printable materials, specialist equipment and significant user expertise, which limits its widespread application.

### 4.5 Other techniques

Overall, above additive manufacturing strategies could achieve the rapid and simple fabrication of continuously graded biomaterials; however, these strategies are generally restricted to simple and unidirectional gradients. In addition to depositing the material directly along the gradient axis at different spatial coordinates, an alternative strategy is to start with a uniform system and redistribute the components into gradients with an applied force, such as buoyancy, magnetic attraction and electric attraction which enable gradients to be formed without needing to modify any of the components.

**Fig. 9:**
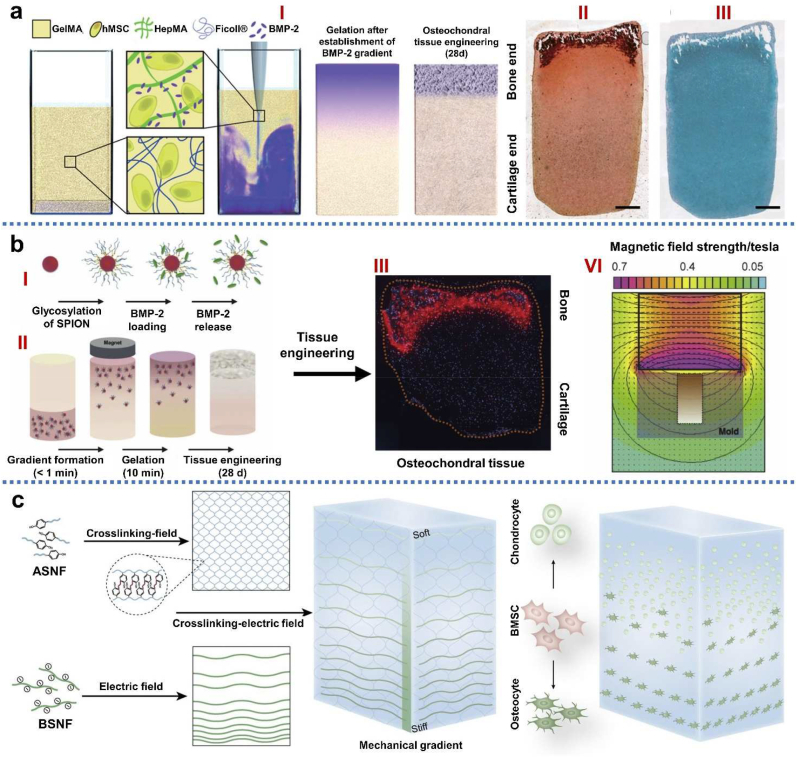
Integrated hierarchical osteochondral scaffold was designed by buoyancy, magnetic attraction and electric attraction techniques. (a) Growth factor gradients for osteochondral tissue engineering: I) Osteochondral tissue, engineered using of hMSC-laden GelMA hydrogels, with buoyancy used to form a morphogen gradient of BMP-2 complexed with heparin methacrylate (HepMA); II) Alizarin Red S staining revealed localized mineral deposition at one end of the tissue; III) Alcian Blue staining revealed tissue-wide staining for glycosaminoglycans, a component of both cartilage and bone [116]. (b) Engineering osteochondral tissue using magnetically-aligned glycosylated SPIONs: (I) SPIONs were conjugated with heparin to produce a glycosylated corona that could efficiently sequester and release growth factors; (II) An external magnetic field was used to field-align glycosylated SPIONs in a hMSC-laden agarose hydrogel, which was thermally gelled and cultured for 28 days to generate robust osteochondral constructs comprising both bone and cartilage tissue; (III) Finite element modeling of the magnetic field strength and distribution; (VI) The key mineralization protein osteopontin (red), which were present specifically at the bone end of the tissue [117]. (c) Using electric field migration to fabricate silk nanofiber hydrogels with gradients and the control of cell differentiation [118]. Reproduced with permission: (a) [116], copyright 2019, Wiley; (b) [117], copyright 2018, Elsevier; (c) [118], copyright 2020, Springer.

Li et al. [116] developed a generalized buoyancy-driven approach to generate tunable transitions in composition, biochemical profile and compressive stiffness by systematically varying the material characteristics and injection parameters. Here, a BMP-2 gradient, presented across a gelatin methacryloyl hydrogel laden with hMSCs, was used to locally stimulate osteogenesis and mineralization in order to produce integrated osteochondral tissue constructs (Fig. 9a). The resulting tissue constructs possessed distinct regions of bone and cartilage, along with a structural transition that resembled the native tidemark in osteochondral tissue. The versatility and ease of use of this fabrication platform offered the opportunities to generate other gradient materials or interfacial tissues.

In 2018, Li et al. [117] used magnetic field alignment of glycosylated superparamagnetic iron oxide nanoparticles, pre-loaded with BMP-2, to pattern biochemical gradients into a range of biomaterial systems. These BMP-2 gradients were formed across agarose hydrogels laden with hMSCs to generate integrated osteochondral tissue constructs. The smooth gradients of BMP-2 gave rise to emergent structural features that highly resembled the osteochondral interface, including a tidemark transition demarcating mineralized and non-mineralized tissue and an osteochondral interface rich in hypertrophic chondrocytes (Fig. 9b). Overall, this platform technology provided a new opportunity for overcoming a series of challenges of interfacial tissue engineering.

In 2020, Xu et al. [118] used electric field migration to fabricate biomaterials with compositional and mechanical gradients. Specifically, β-sheet rich silk nanofibers (BSNF) were used as building blocks to introduce multiple gradients into different hydrogel systems through the joint action of crosslinking and electric field. Here, β-sheet rich silk nanofibers moved to the anode of an applied electric field, with the migration kinetics tuned to the gelation rate of the surrounding polymer (Fig. 9c). The results demonstrated that the hydrogels possessed suitable mechanical gradients to tune osteogenic-chondrogenic capacity, which stimulated the ectopic osteochondral tissue regeneration *in vivo*. The versatility and highly controllability of this strategy broadened its applicability in complex tissue engineering and various interfacial tissues.

Although some achievements have been realized in *in vivo* and clinical reports, almost in all current studies, solely, an incomplete biochemical gradient was created, and gradient in other features and function of the scaffold was lacking. Most importantly perhaps, we need to deeply understand the mechanism and various variation in functional and structural properties of osteochondral defect. Factors such as possible changes in the metabolic pathway (oxygen and nutrients), alterations in the biological remodeling dynamics, and variations in collagen and mineral status and perhaps their spatial distribution are currently under investigation. In terms of clinical effect, differences in age and disease status of individuals established a major challenge in fabricating engineering scaffolds that could meet the demand of specific repair sites in specific patients. Therefore, many challenges still need to be resolved in order to create functional osteochondral scaffolds that could eventually be used in clinical practice.

## 5 Evaluation of IGTEOS

Since it is difficult to compare the scaffolds' results with each other from different scientific papers, there is an urgent need for a system to regulate the scaffolds’ *in vitro/in vivo* test results. By developing universal grading criteria and a specific set of tests with controlled parameters, it would be possible to compare the results of different scaffold designs and help determine which specific features of osteochondral scaffold optimize performance. Currently, IGTEOS has been assessed *in vitro* and toward osteochondral defect animal models *in vivo* to evaluate its safety and effectiveness, which promotes its application possibility in clinical trials. To assess whether fabricated osteochondral scaffolds are viable for osteochondral regeneration, they should be chemically, structurally, mechanically and biologically evaluated. We outlined the evaluation index and methods of osteochondral scaffold in Table 5.

**Table 5:** *In vitro* and *in vivo* evaluation of IGTEOS.

Evaluation		Evaluation index		Evaluation methods
***In vitro***	Interfacial bonding strength	Sufficient bonding strength		Shear testing and peel testing
	Cell compatibility	Cell adhesion and viability		SEM and live/dead staining
	Chondrogenic differentiation	Chondrogenic markers	SOX9	Immunofluorescence staining
			Col-II	
			Aggrecan (ACAN)	
		Chondrogenic gene expression		Real time-PCR (RT-PCR) procedure
		Production of glycosaminoglycan (GAG)		Toluidine blue and sarfranin O staining
	Osteogenic differentiation	Osteogenic markers	RUNX2	Immunofluorescence staining Alkaline phosphatase (ALP) staining
			Osteocalcin (OCN)	
			Alkaline phosphatase (ALP)	
		Osteogenic gene expression		Real time-PCR (RT-PCR) procedure
		Calcium deposition		Alizarin RedS (ARS) staining/von Kossa
***In vivo* (animal model)**	Macroscopic assessment	Gross morphology assessment scores using modified Wayne's grading scale		Based on the degree of defect repair, degree of integration and macroscopic appearance
	Microcomputed tomography (micro CT)	Percentage bone volume over total volume (% BV/TV)		Scanco Medical 40 Micro CT system
	Histological analysis	Repair tissue morphology, composition and arrangement, cell ECM production and scaffold degradation		Hematoxylin and Eosin (H&E) staining
		The presence of proteoglycans		Toluidine blue staining
		Production of Glycosaminoglycans		Safranin-O/Fast green staining
		Collagen		Masson's trichrome staining
		Fibrin and collagen fibres		Movat's pentachrome/Sirius red staining(*in vitro*)
	Immunohistochemistry analysis	Formation of type II collagen		Collagen were incubated with specific antibody
	Biomechanical	Modulus, Permeability, Poisson's ratio		Custom-designed indentation apparatus

### 5.1 Gradient structure, composition and mechanical evaluation

The scaffold micro-architecture could be analyzed using scanning electron microscopy (SEM). Radhakrishnan et al. [62] used SEM to characterize the longitudinal sections of chondral, subchondral and interfacial layers in lyophilized gradient scaffold. The results showed that pore size decreased gradually from the subchondral zone to chondral zone. The osteochondral interfacial region showed distinct variation in porous morphology of two regions with appreciable interpenetration between the layers at the interface. Energy dispersive spectroscopy (EDS) could be further used to determine the local composition of the samples over a depth of a few micrometers at different regions of the samples [58]. To evaluate the architecture, composition, and porosity profiles of the 3D anisotropic and isotropic gradient structures, X-ray computed microtomography (micro-CT) reconstruction and analysis were performed by Canadas et al. [178]. Coronal and transversal sections of the bi-layered structures showed its continuous interface, and the ceramic phase was distributed inside the structure's volume. The qualitative and quantitative analyses of crystalline phases presented on the powders and scaffolds could be obtained by X-ray diffraction (XRD). For example, the formation of nHA was confirmed according to the characteristic peaks of XRD patterns [178]. Fourier transform infrared spectroscopy (FT-IR) could also be used to investigate the secondary conformations of the scaffolds, and show the characteristic spectra of chemical groups of each component [58].

Compressive stiffness, toughness, strength and shock absorption are characteristics of joints, so some mechanical requirements of osteochondral scaffolds must be fulfilled. Gan et al. [81] examined the compressed mechanical properties of scaffolds using an electromechanical universal testing machine. The compressive strength was defined as the point at which lines from the initial linear region and terminal linear region intersected. The elastic modulus was calculated as the ratio of stress to strain or the slope of the initial linear region of a stress versus strain plot, using the initial cross-sectional area in the calculations [179]. The tensile property measurements of scaffolds could be performed using a universal testing machine [81]. The Young's modulus, tensile strength, and ultimate elongation were calculated from the resultant engineering stress-strain curves [180].

### 5.2 Evaluation of interfacial bonding strength of osteochondral scaffold

The high bonding strength demonstrated that osteochondral scaffold could better mimic the heterogeneous features of the natural osteochondral tissue. The bonding strength between.

Subchondral layer and cartilage layer was examined by shear testing and peel testing along with the axial direction of the osteochondral scaffold by wang et al. [80]. The shear testing followed lap shear ASTM D3163 testing. The peel testing followed ASTM D3330 Method A (180° peel). Levingstone et al. [110] reported that interfacial adhesion strength between the layers of the construct was determined using a custom-designed interfacial strength test rig fitted to a Zwick Z050 Mechanical Testing Machine. Scaffold samples were adhered to aluminum test stubs using a high viscosity adhesive and inserted into the rig for testing. The high viscosity of the adhesive used ensured minimal integration into the scaffold. Failure was expected to occur either at the ultimate tensile strength of one of the component layers of the scaffold or as a result of delamination at the layer interfaces.

### 5.3 Evaluation of cells adhesion and viability *in vitro*

Generally, the adhesion, viability and proliferation of cells on osteochondral scaffold were evaluated by SEM, live/dead staining and CCK-8 assay, respectively. Ding et al. [69] used a two-step process to seed adipose-derived stromal cells (ADSCs) onto each layer of the integrated tri-layered silk fibroin scaffold. Specifically, the ADSCs were divided into two groups, and cultured in chondrogenic or osteogenic induction medium, which allowed an adequate number of chondrogenic- and osteogenic-induced ADSCs to be obtained. Then, chondrogenic-induced ADSCs were first seeded onto the chondral layer of the scaffolds, and incubated for 2 h to allow for cell infiltration and attachment before adding fully supplemented media. After 1 day of culturing, the cell-scaffold constructs were inverted, osteogenic-induced ADSCs were seeded onto the bony layer. After some days of culturing, the cell-scaffold constructs were fixed with 2.5% glutaraldehyde and then dehydrated through a graded series of ethanol. The adhesion of cells on each layer of the scaffold was observed by SEM. The viability of the cells within the scaffold was assessed by use of a live/dead viability assay kit, and live cells (green) and dead cells (red) on each layer of the scaffold were observed by confocal microscopy. In the same way, Wang et al. [80] seeded rBMSCs on both sides of the 3D-printing osteochondral scaffolds. A live/dead viability assay kit was used to stain cells to investigate the cyto-compatibility of osteochondral scaffolds. The proliferation of rBMSCs in both cartilage layer and subchondral layer was examined using CCK8 assay.

### 5.4 Assessment of isolating role of the intermediate layer

The calcified cartilage is a transition layer that acts as a physical barrier to inhibit vascular invasion into the cartilage to prevent the ossification of full-thickness cartilage [108]. To confirm that the intermediate layer served as an isolation layer, Ding et al. [68] seeded DiO-labeled cells (DiO, green fluorescent dye) and DiI-labeled cells (DiI, red fluorescent dye) onto the chondral and bony layers, respectively. After some days of culture, the labeled cell-scaffold construct was cut into longitudinal sections. The sections were stained with 4′, 6-diamidino-2-phenylindole (DAPI) and then directly observed. Under a confocal microscope, the DiO-labeled cells (green) only distributed in the chondral layer, whereas the DiI-labeled cells (red) only distributed in the bony layer. The cell-free zone was visible between the chondral layer (green) and the bony layer (red). These results suggested that the intermediate layer plays a role in preventing the cells within chondral and bony layers from mixing with each other.

### 5.5 Evaluation of cell differentiation *in vitro*

The biological properties of scaffold could be evaluated by gene expression methods such as polymerase chain reaction (PCR) and reverse transcription polymerase chain reaction (RT-PCR) as well as histological and immunohistochemical methods. Gradient scaffolds resulted in different cell behavior and tissue formation in different regions. Improved chondrogenic differentiation of cells at the cartilage layer was achieved by showing up-regulated expression of chondrogenic markers (including SOX9, Col-II and aggrecan (ACAN)) and the production of GAG, while enhanced osteogenic differentiation of MSCs at the subchondral layer was obtained by showing up-regulated expression of osteogenic markers (Runt-related transcription factor2 (RUNX2), osteocalcin (OCN), osteopontin (OPN) and alkaline phosphatase (ALP)), and calcium deposition. Briefly, MSCs were included in both layers during the fabrication process of hydrogels, or were seeded onto the chondral and bony layers of scaffolds respectively, and then the cell-scaffold constructs were cultured in osteochondral differentiation medium for 28 days. At the predetermined time points, chondrogenic-osteogenic differentiation of MSCs was identified by immunochemistry double-staining for RUNX2/SOX9, OCN/Col-II, and OPN/ACAN, respectively [118]. Production of GAG, ALP and calcium deposition were labeled *via* histochemical staining. In addition, chondrogenic and osteogenic genes expression levels were further analyzed by real-time quantitative polymerase chain reaction (RT-qPCR) [181]. At the desired time points, total RNA from cells cultured on hydrogels was isolated using total RNA extraction kit. 1 μg of the extracted RNA were transcribed into complementary DNA (cDNA) using a High-Capacity cDNA Reverse Transcription kit. Then, relevant gene expression of each sample was measured by RT-qPCR using Step One Plus real-time PCR system using a SYBR Green rapid assay kit.

### 5.6 Macroscopic assessment of the level of repair at defect sites

Photographs of the defect sites were taken and the quality of cartilage repair and regeneration were assessed blindly by three different assessors using modified Wayne's grading scale in which the score based on the color, defect filling, edge integration and smoothness of cartilage (Table 6). On opening of the joints, gross macroscopic visual evaluation of the repair tissue was carried out. Gross morphological scores were consistent with findings of the visual evaluation.

**Table 6:** Gross morphology scoring by modified Wayne's grading scale [182,183].

Gross appearance	Score-0	Score-1	Score-2	Score-3	Score-4
**Coverage (% fill)**	No fill	<25	25–50	50–75	>75
**Tissue color (% yellow/brown/reddish)**	100	75	50	25	Normal/Whitish
**Surface (smooth level)**	Irregular >75%	Irregular 50–75%	Irregular 25–50%	Smooth but raised	Normal
**Defect margins (circumference visible)**	Entire	75%	50%	25%	Invisible

### 5.7 Micro-computed tomography evaluation of subchondral bone formation

To obtain both qualitative and quantitative measurements of the new bone regeneration level within the osteochondral defect site, Micro-CT analysis could be performed to scan the medial femoral condyles in the scaffold-implanted and empty defect groups after about 4 and 12 weeks of post-implantation. The dissected rabbit femur ends were fixed with 10% neutral-buffered formalin fixation, and then were loaded on a sample holder with the femur axis perpendicular to the scanning plane. Skycan packaged software (including Skyscan CT-Analyzer program v.1.8, CT-Volume v.2.0 and Data Viewer), Image-J and Bone-J software were used to reconstruct the image data and visualize the representation of the newly formed bone. To quantify the amount and quality of the newly formed mineralized tissue and the residual materials, the region of interest (ROI) 3 mm in diameter within the repaired site was chosen with three dimensional reconstructions using the Mic View software to distinguish the newly formed bones and the residual materials. Subchondral bone repair (volume and diameter of the bone growth) was expressed as percentage bone volume over the total volume (% BV/TV), trabecular thickness (Tb. Th), and bone mineral density (BMD).

### 5.8 Microscopic and histological assessment of the level of repair at defect sites

Histological staining analysis further confirmed that the prepared scaffold was able to simultaneously enhance the repair of articular cartilage and subchondral bone, relative to the untreated control. A limited number of stains was sufficient to provide a thorough evaluation of the gross histomorphology of osteochondral defect repair sites. Histological sections specimens of osteochondral from medial femoral condyles were obtained by standard process, including 10% neutral-buffered formalin fixation, gradient dehydration, decalcification, sectioning perpendicular to the longitudinal axis, infiltration and paraffin embedding. Following dewaxing, sections were stained histologically following standard protocols in order to assess, the quantity and quality of repair tissue and integration with native tissue. Hematoxylin-eosin (H&E) for an overview of the tissue section (including cell arrangement and morphology, tissue formation and integration, ECM production and scaffold degradation), and the tidemark visibility was influenced by the choice of hematoxylin. The toluidine blue staining was used to assess the presence of proteoglycans, to get an overview of the tissue structure with a high contrast. Safranin-O with fast green counterstain was used to assess the presence of GAG within the repair tissue in the cartilage region. Alcian blue, a cationic water-soluble dye, selectively stained sulfated GAG at low pH (∼1.0). Collagens could be stained with either Masson's trichrome, Mallory trichrome, or Sirius red. Masson's trichrome staining could identify content and alignment of collagen and Movat's pentachrome could identify fibrin and collagen fibers. One could choose between the safranin O and the Alcian blue stain, as well as between the toluidine blue and H&E stain. Images from each specimen were acquired using standard bright-field and polarized light microscopy and digital images captured [166,184]. Notably, tissue sampling and preservation should be carried out by fast processing and the use of cationic dyes in the fixation solutions to prevent the loss of proteoglycans. Thus, the GAG content of the neo-cartilage and adjacent cartilage was commonly assessed using Alcian blue staining [185]. The stained sections under polarized light could show the orientation of the collagen fibers, which was one of the easiest ways to distinguish hyaline cartilage from fibrocartilage and revealed whether the repaired tissue was continuous with the subchondral bone. Furthermore, Col-I and Col-II deposition was evaluated through standard immunohistochemistry. Compared to the empty defect group, adequate healing of the osteochondral defect was seen in the scaffold implanted group, with the regeneration of bone tissue in the subchondral region and the formation of an overlying cartilage layer.

Semi-qualitative histological scoring could be carried out independently by a certified histopathologist under blinded conditions using the histological scoring system based on the modified O'Driscoll system and the Holland's scoring system [62,150,182,186]. This histological scoring system has been previously validated for the assessment of articular cartilage repair while also allowing assessment of the subchondral bone. The GAG content of the neo-cartilage and adjacent cartilage was graded from 0 to 3 using Alcian blue staining [185]. Histological assessment of repair should support the macroscopic and micro-CT findings with defect repair occurring in the scaffold implanted group.

### 5.9 Biomechanical evaluation

Substantial changes in osteochondral mechanical properties occur during the development of osteoarthritis, which provide an effective way to evaluate the therapeutic effect of osteochondral scaffolds.

#### 5.9.1 Biomechanical indentation testing

The animals were sacrificed at 3 and 6 months postoperatively, and the knee joints were exposed by a medial parapatellar arthrotomy. Immediately, indentation testing could be performed using a custom-made high-precision materials testing equipment to determine the mechanical properties of the healthy and transplanted cartilage in indentation [187]. LVDT displacement transducer and a load cell were used to record displacement and force. Each sample was tested with a porous spherical indenter perpendicular to the joint surface at the center of the defect. The stress-relaxation/creep behavior of each cartilage sample was recorded as a function of time. Biomechanical evaluation was accomplished on the basis of four parameters: Modulus, Permeability, Poisson ratio. The elastic modulus and the aggregate modulus of each sample were calculated from the linear range. The dynamic modulus were calculated from the peak stresses and the amount of deformation at each step. The Poisson ratio was calculated by indentation with different indenter sizes, and the permeability was calculated by finding best-fit approximations for relaxation behavior of two-phase model. Shi et al. [188] reported that biomechanical analysis of rat cartilage tissue were carried out using an *in situ* nanomechanical test system. PBS solution was used to maintain cartilage hydration. The indentation cycle consisted of a 10 s peak load, 2 s hold, and another 10 s unload. The maximum indentation depth was 2000 nm. Hardness and elastic modulus were determined from the load-depth curve.

#### 5.9.2 Biomechanical push-out test

The interfacial strength of neo-tissue integration with host could be evaluated by a biomechanical push-out test. The excised bone tissue consisting of native and neo-tissue at −80 °C were thawed slowly in airtight tubes and sliced to 3 mm thickness. The tissue sample was mounted in a customized fixture setup and then push-out on a uniaxial mechanical tester equipped with a 500 N load cell. The indenter of push-out setup was allowed to pass through the defect site at a constant rate of 0.5 mm/s until the material failure. Failure loads were temporarily monitored as the new tissue was pushed out. The maximum load and interfacial shear stress of the four groups were recorded. Maximum load required for failure directly relates to interfacial bond strength between the newly regenerative tissue and the adjacent host tissue [62]. Additionally, the mechanical strength may be associated with the significantly higher mineralization of the subchondral bone [157].

## 6 Perspectives towards future development

Considering that osteochondral tissue has a stratified structure because of the required unique properties, osteochondral scaffolds need to have multiphase structures to simulate the structure of the native stratified tissue. However, due to the complexity of the multiphasic scaffolds, it is difficult to control the performances of each phase, such as degradation rate, mechanical stability, etc. Especially, shear forces between different phases of the scaffold would not only lead to the interfacial stress concentrations and decreased integration, but also weaken the long-term durability after implantation [[189], [190], [191]]. To effectively minimize the above adverse influences, using a single technique to fabricate the whole IGTEOS should be one of worth-considering strategies. Electrospinning may be one such appropriate candidate technique, by which different parts with different compositions could be combined together in the IGTEOS. Importantly, strong combination of two adjacent phases in the scaffold could be hopefully realized by an electrospun conjugating layer made from mixture of the two-phase materials. We have obtained satisfactory combining effect of two different parts in one scaffold using the related technique in our previous study [192]. Besides, 3D printing may be another properly fitting technique to prepare IGTEOS, which could not only fabricate the scaffold with different composition at its different parts by using various ink, but also precisely control the complex hierarchical structure by applying well-determined parameters based on the specific requirements of bone and cartilage regeneration. Moreover, it is possible to architect the scaffold by 3D printing to control the stress distribution and resist the external force as well as possible, thereby benefiting cell behaviors and tissue regeneration [177].

As for selection of the composition in IGTEOS, although biomimetic method should be given priority, it has always been difficult to obtain totally biomimetic raw materials, such as collagen, from the manufacturers because some chemical groups and microstructures could be nearly unavoidably changed during the current process. In many cases, those changes could have significant negative influence on the final biomimetic efficacy, which however has not aroused enough attention to date. There may be two main means that should be developed further to improve the above situation. One is reprocessing the purchased materials by some specific methods, such as self-assembling, recrystallizing, etc. The other is optimizing current techniques or developing new approaches to keep natural characteristics of the materials as well as possible during the processing.

Further, since the load of growth factors or genes is one of main means to enhance the bioactivities, the development of ready-to-use high-performance IGTEOS for delivering those bioactive substances should be another indispensable effort point. Given that the final goal of IGTEOS is to facilitate the regeneration of both bone and cartilage, it should be emphasized that at least two kinds of bioactive substances could be properly loaded into different parts of the scaffold and could be all released in a sustained fashion. Different bioactive substances possess different physicochemical structures. So specific studies should be launched to control the structure of different parts in the scaffold according to the specific structure of the respective ready-to-load bioactive substances to supply satisfactory physical combination, and/or to functionalize the scaffold with specific chemical groups at different layers to realize appropriate chemical interactions with the different bioactive substances.

On the other hand, it has been well recognized that an appropriate magnitude of various external physical cues such as mechanical stimulation, electrical stimulation, and magnetic stimulation, can enhance specific cellular and tissue behaviors. Therefore, it should be one promising research direction to develop special IGTEOS with adequate consideration of being able to effectively utilize those external physical cues. For the use of mechanical stimulation, besides meeting the requirement of suitable stiffness and morphology, the scaffold should possess appropriate capability of mechano-transduction. Hydrogels may be one kind of ideal materials used to prepare the IGTEOS in this aspect due to their effective mass transfer. For the utilization of electrical stimulation, it is usually demand that the scaffold have certain ability of electric conduction. There are normally two approaches to fabricate electrically conductive scaffolds, one of which uses intrinsically conductive materials while the other incorporates conductive materials into a non-conductive matrix. Carbon nanotubes, which possess excellent electroconductivity and have presented big potential to be used as scaffold materials, may be one kind of satisfactory candidate additives in the IGTEOS to bring adequate electroconductivity. For the employment of magnetic stimulation, it is recommended that magnetic nanoparticles (MNPs) are integrated into the IGTEOS through diffusion, simple mixing or electrospinning a mixture of matrix solution and MNPs.

Finally, it is urgent to further develop the *in vitro* bio-functional evaluation systems and methods for the IGTEOS, which will shed light on targeted strategies to better optimize the scaffold. Since satisfactory IGTEOS needs to support and promote both bone and cartilage regeneration, it is necessary to figure out how to better qualitatively and quantitatively evaluate the capacity of IGTEOS in inducing osteogenic and cartilaginous differentiation of cultured cells in one system, and make more clear the details about the interactions between the clues in the IGTEOS promoting cellular osteogenic differentiation and those inducing the cartilaginous differentiation, and even reveal the related underlying biochemical mechanisms.

## 7 Conclusions

The field of tissue-engineered osteochondral scaffold has grown steadily over the past ten years. In this review, we brought forward the main challenges of establishing a satisfactory IGTEOS, and discussed the current tissue-engineered efforts to resolve the above challenges, including tissue-engineered strategies and evaluation methods of IGTEOS. Considering the current situation of osteochondral defect repair, a number of formidable challenges remain within the domain of osteochondral tissue engineering. None of the studies reviewed here have created a tissue that fully regenerates the natural zonal organization of osteochondral tissue, even after several months *in vivo*. Based on the current challenges and research progress, we further analyzed in details the future perspectives of tissue-engineered osteochondral construct. Perhaps, the success of osteochondral regeneration in the future will depend on the convergence of scaffold, gene delivery technologies, various external physical cues and excellent evaluation systems. A final hurdle to full osteochondral regeneration is the integration of any neo-tissue with the existing tissue. In order to achieve tangible and clinically relevant results, a sustained collaborative effort from all fields in the tissue engineering domain will be required, focusing on the biology of the bone and cartilage tissue systems.

## Ethics approval and consent to participate

I confirm that I have obtained all consents required by applicable law for the publication of any personal details or images of patients, research subjects or other individuals that are used in the materials submitted to KeAi. I have retained a written copy of all such consents and I agree to provide KeAi with copies of the consents or evidence that such consents have been obtained if requested by KeAi.

## Declaration of competing interest

There are no conflicts of interest to declare.
